# Probing Passive Permeation of Tetracycline: Are Simulations Ready for beyond-Rule-of-Five Drug Permeability Calculation?

**DOI:** 10.1021/acs.jpcb.5c05445

**Published:** 2025-10-03

**Authors:** Yajing Qi, Christophe Chipot, Yi Wang

**Affiliations:** † Department of Physics, 26451 The Chinese University of Hong Kong, Shatin, Hong Kong SAR, China; ‡ Laboratoire International Associé Centre National de la Recherche Scientifique et University of Illinois at Urbana-Champaign, Unité Mixte de Recherche no 7019, Université de Lorraine, 54506 Vandœuvre-lès-Nancy, France; ¶ Theoretical and Computational Biophysics Group, Beckman Institute, and Department of Physics, University of Illinois at Urbana-Champaign, Urbana, Illinois 61801, United States; § Department of Biochemistry and Molecular Biology, University of Chicago, Chicago, Illinois 60637, United States

## Abstract

Passive permeation across lipid membranes is a key determinant of drug bioavailability and efficacy. Accurate computational estimation of drug permeability is essential for rational drug design, yet remains challenging, particularly for ionizable and beyond-Rule-of-Five (bRo5) compounds. In this study, we employ advanced molecular simulations and the inhomogeneous solubility-diffusion model to calculate the effective permeability and elucidate the membrane permeation mechanism of the antibiotic tetracycline (TC), the six hydrogen-bond donors of which violates one of Lipinski’s Rule-of-Five. By integrating the pH-partitioning and Boltzmann-weighted average potential schemes and accounting for both its neutral (TC_N_) and zwitterionic (TC_Z_) tautomers, we show that the dominant contribution to the effective permeability of TC arises from TC_N_, despite its low abundance. This result is attributed to the relatively small permeation barrier of TC_N_, explaining why the antibiotic exhibits moderate effective permeability even though it predominantly exists in the zwitterionic form at neutral pH. A further systematic investigation reveals that membrane patch size significantly impacts permeability estimates for TC, in contrast to the relative insensitivity observed for three other permeants. This unique sensitivity can be attributed to the hydrogen-bond network formed between TC and its lipid environment, with the smallest 32-POPC patch artificially raising the drug molecule’s permeation barrier and the largest 256-POPC patch exhibiting significant hysteresis that compromises the quality of the one-dimensional free-energy calculation. Overall, our results suggest that while probing the passive permeation of bRo5 drugs by molecular simulations appears increasingly feasible, the protonation and tautomeric states of the permeants, the uncertainty in their microscopic acid dissociation constants, as well as the potential impact of membrane patch-size effects need to be fully considered in permeability predictions for these complex molecules.

## Graphical abstract

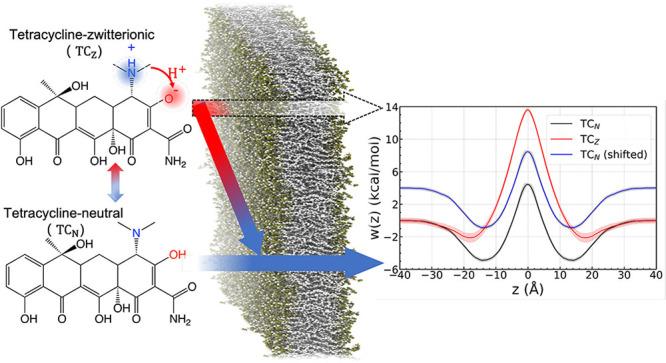

## Introduction

The passive permeation of drug molecules across lipid membranes critically influences their bioavailability and therapeutic efficacy.^1^ Gaining molecular-level insight into how small molecules traverse lipid bilayers and quantifying the associated membrane permeability is key to the rational design of drugs with desirable ADMET (adsorption, distribution, metabolism, excretion, and toxicology) properties.^2^ Experimentally, such permeability is often estimated from the flux across a monolayer of cells (Caco-2) or through the parallel artificial membrane permeability assay (PAMPA).^3–5^ Computationally, molecular dynamics (MD) simulations are frequently employed to investigate the thermodynamic and kinetic underpinnings of passive permeation.^6–9^ While alternative approaches have yielded notable successes,^10,11^ the majority of these simulations have been based on the “inhomogeneous solubility-diffusion” (ISD) model,^12,13^ which combines the potential of mean force (PMF) underlying the translocation of a permeant molecule across a lipid bilayer with its position-dependent diffusivity profile to yield the membrane permeability (*P*):

$$\frac{1}{P}=\int_{z_{1}}^{z_{2}}dz\frac{e^{+\beta w\left(z\right)}}{D\left(z\right)}$$ 1

where the membrane is assumed to span over [*z*_1_, *z*_2_] with its normal placed along the *z*-axis; The PMF *w*(*z*) measures the free energy profile as a function of the permeant molecule’s *z*-coordinate, while *D*(*z*) stands for its position-dependent diffusion coefficient; β = (*k*_B_ *T*)^−1^, with *k*_B_ representing the Boltzmann constant and *T* the temperature. Based on eq 1, permeability calculations from molecular simulations have been performed on a variety of drug or drug-like small molecules.^10,11,14–19^ Deviations of calculated permeabilities from the corresponding experimental references have been found to be as low as 0.12 log unit,^19^ although deviations greater than one log unit are frequently reported.^9,11,17,20^ A major contribution to errors in the computed permeabilities is the PMF calculation—since *w*(*z*) appears in the exponent of eq 1, even small errors in the PMF can be amplified, resulting in significant uncertainty in the calculated permeability.

For ionizable molecules, an additional complexity arises in their permeability calculation: With the exception of constant-pH treatment,^21^ molecular simulations are conducted for a single ionization state of the permeant. For example, an acid molecule with one ionizable group (HA *⇌* H^+^ + A^–^) has two ionization states. A molecular simulation containing either the negatively charged species A^–^ or the neutral species HA can only produce the PMF and diffusivity profiles for that species. The respective permeability obtained via eq 1, termed *specific* permeability,^18^ reflects the intrinsic membrane-crossing capacity of the permeant in the given ionization state, and, by definition, is pH-independent. The combined permeability of all ionization states of a given molecule, termed the *effective* permeability, is pH-dependent and needs to be estimated from the PMF and diffusivity calculations of its individual ionization states.^22^ Recently, Harris et al.^18^ thoroughly evaluated two such protocols, namely, the pH-partitioning and the Boltzmann-weighted average potential (BWAP). In the former scheme, following the pH-partition hypothesis, the permeant molecules in bulk solution are assumed to be an equilibrium mixture of each ionization state, with the fraction of a given state determined by the Henderson–Hasselbalch equation. *P*_*s*_, the specific permeability for each ionization state *s*, is calculated using eq 1 from the corresponding PMF and diffusivity, *w*_*s*_(*z*) and *D*_*s*_(*z*), respectively, and then combined to yield the effective permeability:^18^

$$P_{\mathrm{eff}}=\underset{s}{\sum }f_{s}P_{s}$$ 2

where *f*_*s*_ represents the fraction of the ionization state *s* at the given pH. The pH-partitioning protocol assumes that the protonation/deprotonation transitions of the permeant are much slower than its diffusion along *z*, i.e., the molecule permeates the membrane before it has time to switch its ionization state. In contrast, permeability calculation via BWAP, which underlies the constant-pH treatment,^21^ assumes that the protonation/deprotonation transitions are much faster than diffusion. As a result, an equilibrium between each ionization state is always established along *z*, and the permeant diffuses on a BWAP, *w*_m_(*z*), given by^18^

$$e^{-\beta w_{m}(z)}=\sum_{s}f_{s}e^{-\beta w_{s}(z)}$$ 3

This BWAP, along with an effective diffusion coefficient (eq 17 of Harris et al.^18^), is then used to compute *P*_eff_ of the ionizable permeant via eq 1. In general, whether the pH-partitioning or BWAP protocol should be employed to calculate *P*_eff_ depends on how fast the permeant crosses the membrane relative to its protonation/deprotonation. For drug molecules with a high effective permeability, such as nicotine and varenicline at neutral pH, the pH-partitioning scheme has been found to better characterize the physics of their permeation processes.^18^

The importance of considering protonation/deprotonation kinetics has also been discussed recently by Sezer and Oruç^23^ in their analysis of liposomal fluorescence assays designed by Eyer et al.^24^ to measure the permeability of drug molecules that are either weak acids or weak bases. Compared with PAMPA and Caco-2 assays, these liposome-based assays have negligible unstirred water layer (UWL) effect^25^ and no paracellular contributions, making the measured permeability easier to interpret.^18^ In addition, they utilize single-component bilayers (e.g., POPC), allowing the measured *apparent* permeability (*P*_app_) to be directly compared with the computed *P*_eff_ from molecular simulations conducted with the same lipid composition.

Based on liposomal fluorescence assays, Krämer et al.^26^ and Hermann et al.^27^ investigated the permeability of a series of drug molecules, including, notably, some beyond-Rule-of-Five (bRo5) ones. Formulated by Lipinski and colleagues in 1997,^28^ the Rule-of-Five (Ro5) predicts that poor absorption or permeation is more likely when a molecule has one or more violations of the following: no more than five hydrogen bond donors, no more than ten hydrogen bond acceptors, a molecular weight less than 500 and a calculated logarithm of partition coefficient (log P) no greater than five. The Ro5 was set such that among the thousands of drugs in the USAN library investigated by Lipinski et al., there were about 10% outliers for each of the above rules.^28^ The percentage of breaking the “no more than five hydrogen bond donors” rule has been found to be the lowest,^26^ and orally dosed drugs approved over the past several decades each have ∼2 hydrogen bond donors on average.^29^ While compounds violating one or more Ro5 are generally expected to have poor absorption or permeation, exceptions do exist—notably the widely used antibiotic tetracycline (TC). TC and its analogs can treat a broad range of bacterial infections,^30^ some of which have also been shown to inhibit the growth of drug-resistant *M. tuberculosis* .^31^ With six hydrogen-bond donors, TC has one Ro5 violation. The drug molecule has four fused rings and three ionizable groups (Figure 1A,B). Using the aforementioned liposomal assays, Krämer et al.^26^ demonstrated that TC has a moderate permeability at pH = 6 (*P*_app_ = 10^–5.86^ cm/s). At this pH, TC primarily exists as a zwitterion,^32^ i.e., the molecule carries zero net charge, but contains both negatively charged and positively charged groups. While the measured *P*_app_ is consistent with previous results of a PAMPA assay, as well as the fast adsorption and good bioavailability of the drug in vivo,^33,34^ the charged groups carried by the zwitterionic drug have been suggested to significantly reduce its membrane permeability at neutral pH.^35^ Thus, the origin of the moderate membrane permeability to TC remains unclear.

**Figure 1:**
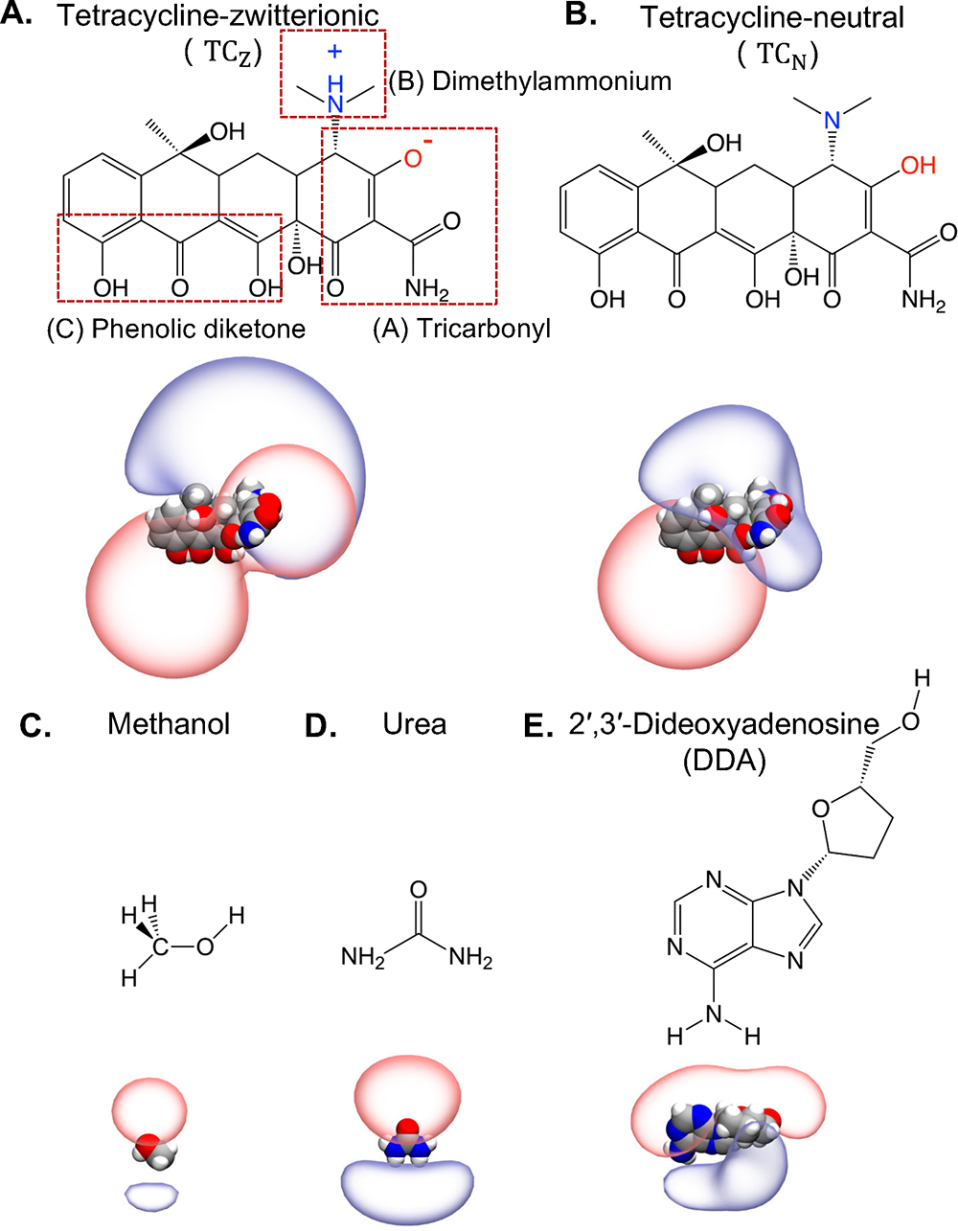
Structural representations of the permeants investigated in this work. The top part of each panel (A–E) shows their chemical formula, while the bottom part shows their three-dimensional structures rendered with ±5 *k*_B_ *T*/*e* electrostatic potential isosurfaces based on atomic partial charges.

In this work, we set out to explore the membrane permeation mechanism of TC through computing its effective permeability. At pH = 6, two important tautomers of the zero-net-charge TC are the uncharged neutral form (TC_N_) and the zwitterionic form (TC_Z_), the latter of which is obtained from the former through transferring a proton from the tricarbonyl group A to the dimethylammonium group B (Figure 1A,B). Through determining the PMFs and diffusivity profiles for both TC_Z_ and TC_N_, we show that unlike previously hypothesized,^36^ an effective “cancellation of charge” produced by the positively charged dimethylammonium and the negatively charged tricarbonyl of TC_Z_ does not grant this tautomer a significant membrane permeability. Instead, the effective permeability of TC in a wide range of pH values is predominantly attributed to the TC_N_ tautomer, despite its much smaller population than TC_Z_. Only by considering both zwitterionic and neutral tautomers in the framework of pH-partitioning or BWAP, a properly weighted *P*_eff_ can be obtained, which falls within approximately one log unit of *P*_app_ measured experimentally.

With its six hydrogen-bond donors, significant membrane deformation is recorded during the permeation of TC. This observation further prompts us to conduct a systematic evaluation of bilayer patch size on the membrane permeability calculation of TC as well as three other permeants varying in their size and polarity, namely, methanol, urea and 2′,3′-dideoxyadenosine (DDA). The influence of the patch size on membrane properties—including lipid diffusion, bilayer structure, and elastic moduli—has been well documented.^37–39^ Smaller membrane patches, often necessitated by computational constraints, can artificially suppress long-wavelength fluctuations, alter lateral pressure profiles, decrease lipid and peptide diffusion, thereby, potentially biasing the permeability estimates. Despite these previous studies, the sensitivity of permeability calculation to bilayer patch size remains underexplored, particularly for drug or drug-like molecules. Through comparative simulations in POPC bilayers with 32, 64, 128, or 256 lipids, we quantify how patch size modulates the thermodynamic barriers associated with membrane permeation of the aforementioned four molecules. Notably, we find that only TC demonstrates a strong patch size dependence in its computed permeability, where the smallest 32-POPC bilayer presents artificially large barriers against its permeation, while the biggest patch with 256 POPC shows sampling hysteresis that compromises the quality of the free-energy calculation using the drug molecule’s *z*-position as the sole collective variable. We attribute this sensitivity to the strong hydrogen-bonding capacity of TC, a hallmark of its Ro5 violation.

Collectively, our results suggest that while permeability calculations for bRo5 drugs based on molecular simulations and the ISD model are increasingly feasible, care must be taken to ensure that (1) for ionizable molecules that can exist in both zwitterionic and neutral forms, both tautomers are simulated and their results combined with other ionization states using the pH-partitioning or BWAP protocols, (2) the bilayer patch size is big enough to avoid artificially suppressing membrane deformation, and (3) potential hysteresis is duly monitored, especially for molecules with strong hydrogen bonding capacity translocating across a large bilayer. Below, we describe our computational methods and results in detail, followed by a discussion on challenges facing bRo5 drug permeability calculation. We note that apart from errors in free-energy and diffusivity calculations, uncertainty in the microscopic p*K*_a_ values can contribute significantly to deviations between computed *P*_eff_ and experimentally measured *P*_app_. In particular, this issue is exacerbated if incorrectly assigned macroscopic p*K*_a_ values are used in place of the appropriate microscopic ones.

## Methods

All MD simulations reported in this work were performed in 1-palmitoyl-2-oleoyl-*sn*-glycero-3-phosphocholine (POPC) bilayers. Unless otherwise specified, a POPC bilayer with 128 lipids was used by default. Three other bilayer patch sizes, namely, 32, 64, and 256 POPC, were subsequently used to examine the effect of patch size on permeability calculation. All bilayer systems were built using the CHARMM GUI program.^40^ For bilayers with 32, 64, 128, and 256 POPC, the total number of atoms was approximately 12,000, 21,000, 39,000, and 81,000, respectively, while the system sizes were approximately 34 × 34 × 105 Å^3^, 48 × 48 × 95 Å^3^, 66 × 66 × 90 Å^3^, and 95 × 95 × 90 Å^3^, respectively. All MD simulations were carried out using NAMD 3.0,^41,42^ with the CHARMM36 force field (FF) for lipids and TIP3P for water.^43,44^ The force field parameters for methanol, urea, and 2′,3′-dideoxyadenosine were obtained from the CGenFF web server.^45,46^ Parameters of TC were taken from Aleksandrov and Simonson,^47,48^ in which parameters compatible with the CHARMM FF were developed for tetracycline and 11 of its analogs. The system temperature was maintained at 310 K using a Langevin thermostat with a damping coefficient of 1 ps^–1^,^49^ with the pressure regulated at 1.01325 bar via the Langevin piston method.^50^ Long-range electrostatic interactions were evaluated employing the particle-mesh Ewald algorithm with a grid spacing of 1 Å,^51^ whereas short-range van der Waals (vdW) and electrostatic interactions were truncated at a smoothed 12 Å cutoff. The vdW potential was treated with a force-switching protocol. Multiple time-stepping was used to integrate the equations of motion, with time-steps of 2 fs for short-range and 4 fs for long-range interactions.^52^ Water molecules were constrained to their equilibrium geometry using the SETTLE algorithm,^53^ and covalent bonds involving hydrogen atoms were constrained with the RATTLE algorithm.^54^

To determine the free-energy profile associated with the membrane permeation of a given molecule, we employed the Well-Tempered Metadynamics- extended-system Adaptive Biasing Force (WTM-eABF) algorithm^55,56^ with the collective variable (CV) defined as the *z*-projection of the center of mass of the permeant. The center of mass of lipid phosphorus atoms was placed at *z* = 0. The overall range of sampling, set to −43 < *z* < 43 Å (−38 < *z* < 38 Å for systems with 256 POPC), was divided into three windows. Each window spanned 28–33 Å, with a 5 Å overlap between adjacent windows. During the WTM-eABF calculation, sampling data were accumulated and recorded using a bin width of 0.2 Å for each window. To minimize spurious nonequilibrium phenomena, the biasing potential was withheld until 20,000 sampling steps had been collected in a given bin.^57^ For the WTM component, the bias temperature was set to 3000 K, with a Gaussian hill width of 5.0 Å and a hill height of 0.1 kcal/mol. For the eABF component, the standard deviation between the colvar and the fictitious particle was 0.05 Å, with an oscillation period of 100 ns.

After obtaining a well-converged PMF, we evaluated the diffusivity of a permeant by applying its inverse PMF in the *z* direction. As a result of the applied inverse PMF, the permeant was able to diffuse freely over an approximately flat free-energy surface along the membrane normal. This was achieved by initializing an ABF calculation with the final, converged PMF, disabling further updates of the biasing force. Since diffusion in the (*x*, *y*)–plane was inherently unrestrained, introducing the reverse biasing force in the *z*-direction allowed for free diffusion in the entire simulation box. The position-dependent fractional diffusivity, as well as the classical diffusivity, were estimated using a variant of the Bayesian inference scheme developed for classical diffusion.^58^ These calculations were performed with the DiffusionFusion program,^59,60^ employing a grid spacing of 1 Å. Finally, analysis of permeant orientation, hydrogen bonds, and the number of water molecules accompanying the permeants in the region −15 Å ≤ *z* ≤ 15 Å was performed using VMD.^61^ The average values and standard deviations for each property were computed for every 1-Å bin along the *z*-axis. Hydrogen bonds were determined using a cutoff distance of 3.6 Å and an angle of 30°. The number of water within 2.4 Å of the permeants was also computed.

## Results

### Specific Membrane Permeabilities to TC_N_ and TC_Z_ Differ by 6 Orders of Magnitude

We begin our investigation of TC membrane permeation by computing the free-energy profiles of two zero-net-charge TC tautomers (Figure 1) crossing a bilayer with 128 POPC lipids, using the WTM-eABF approach. Given that all bilayers in our simulations contain the same number and type of lipids in its two monolayers, the resulting PMFs are symmetrized (see Methods), with the differences between the original, unsymmetrized PMFs and the final, symmetrized ones shown as error bars in Figure 2. All simulations are continued until the corresponding error bars are reduced to approximately 1 kcal/mol or less. The sampling quality is further evaluated based on the degree of convergence of the computed PMFs and their gradients: As shown in Figure S1, the PMFs obtained with increasing simulation time offer a direct assessment of the evolution of the free-energy profiles, where sampling convergence is reflected in the small (<0.5 kcal/mol) differences between the PMF profiles computed using the last ∼0.5-μs simulations. With the final PMF gradient as reference, we also compute the root-mean-square deviation (RMSD) of the PMF gradients obtained with increasing simulation time, the evolution of which (Figure S1) further confirms the convergence of our free-energy calculations. Next, the diffusivity profiles of the two TC tautomers are determined from additional sets of 4- and 2-μs simulations with their inverse PMFs applied as biasing potentials, respectively. The permeants experience an approximately flat free energy surface in these simulations, enabling them to undergo free diffusion along the membrane normal. The position-dependent classical diffusivity profiles computed from the simulation trajectories are illustrated in Figure 2B, while the fractional diffusivity profiles are shown in Figure S2. As with the PMFs, the error bars in Figures 2B and S2 represent the differences between the original, unsymmetrized diffusivity profiles and the ones after symmetrization. It is noteworthy that the diffusivity profiles obtained with the classical and fractional Smoluchowski equations are nearly identical, revealing no hallmark of anomalous diffusion,^62^ which is suggestive that the issue of time scale separation of the CV (projection along the *z*–axis) and the slow degrees of freedom of nearby lipids—or the incompleteness of the CV subspace describing the permeation event—may be less acute for large permeants.^17,59^ Finally, using the ISD model as given in eq 1, the specific permeability of the neutral TC_N_ (*P*_N_) and the zwitterionic TC_Z_ (*P*_Z_) are determined from their respective *w*(*z*) and *D*(*z*) profiles: *P*_N_ = 2.22 × 10^–2^ cm/s and *P*_Z_ = 1.63 × 10^–8^ cm/s. From hereon, we frequently refer to the 10-based log values of the permeabilities (log *P*_N_ = −1.65 and log *P*_Z_ = −7.79) with their units (cm/s) omitted for simplicity.

**Figure 2:**
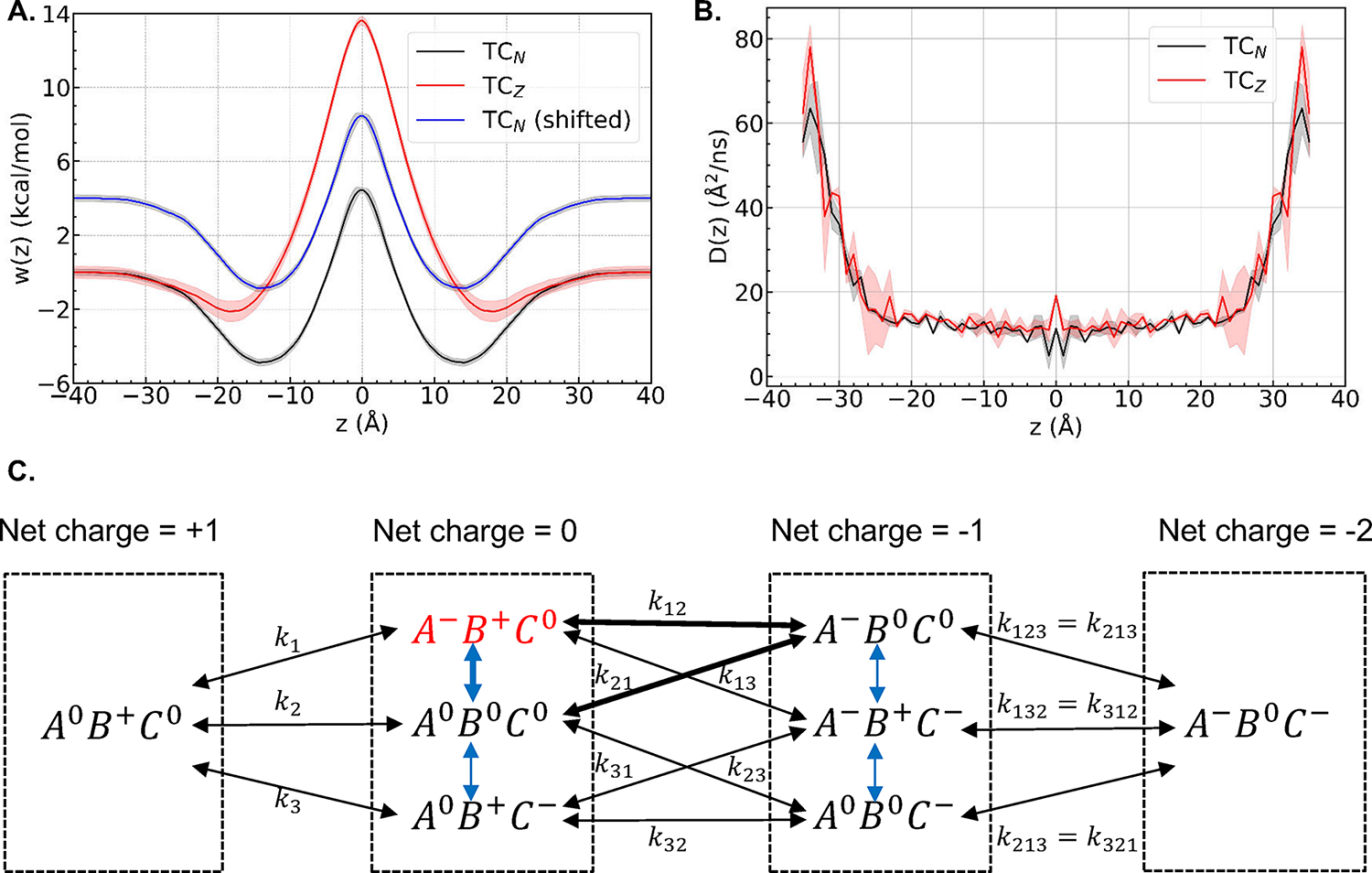
(A) Symmetrized PMFs *w*(*z*) and (B) classical diffusivity profiles *D*(*z*) of TC_N_ and TC_Z_. PMFs for TC_N_ both with and without the free energy shift Δ*G* = −*k*_B_ *T*ln­(*f*_N_/*f*_Z_) are shown. (C) Complete ionization scheme of TC. Black arrows correspond to microscopic p*K*_a_s, and blue arrows indicate the transition between neighboring tautomers with the same net charge. One thermodynamic cycle involving *A* ^–^ *B* ^+^ *C* ^0^, *A* ^–^ *B* ^0^ *C* ^0^, and *A* ^0^ *B* ^0^ *C* ^0^ is highlighted.

Our results show that *P*_N_ is approximately 6 orders of magnitude greater than *P*_Z_, indicating that while both tautomers carry zero net charge, the neutral TC_N_ is considerably more membrane permeable than the zwitterionic TC_Z_. As their diffusivity profiles are nearly identical, the large discrepancy in their specific permeabilities arises almost exclusively from their distinct permeation free energy profiles. While TC_N_ and TC_Z_ both have “W-shaped” PMFs, as observed in the membrane translocation PMFs of many other small molecules,^6,15,20,63^ the permeation energy barriers encountered by the two tautomers differ significantly: the zwitterionic TC_Z_ faces a barrier of 13.6 kcal/mol at the bilayer center (*z* = 0), while the neutral TC_N_ has a barrier of only 4.5 kcal/mol at the same location. Since *w*(*z*) appears in the exponent of the integrand in eq 1, the 9.1 kcal/mol difference in their energy barriers results in the six orders-of-magnitude difference in the specific permeabilities of the two tautomers.

To understand the origin of their distinct permeation free energy profiles, we analyzed the orientations of TC_N_ and TC_Z_ using two vectors: ${\vec{v}}_{1}$ pointing from atom C2 near the tricarbonyl group to atom C11 on the aromatic ring D, and ${\vec{v}}_{2}$ pointing from atom C15 near the phenolic diketone group to atom C22 of the methyl group attached to ring C (Figure 3 A). The position-dependent profiles of their angles with the *z*-axis reveal distinct orientations of the two tautomers (Figure 3B,C): For TC_N_, ${\vec{v}}_{1}$ maintains an approximately 90° angle to the *z*-axis, while the angle between ${\vec{v}}_{2}$ and the *z*-axis switches from ∼150 to ∼30° as TC_N_ crosses the bilayer center, indicating a flip-flop transition along ${\vec{v}}_{2}$ . In contrast, for TC_Z_, ${\vec{v}}_{2}$ maintains an approximately 90° angle with the membrane normal, while a flipflop transition is observed along the vector ${\vec{v}}_{1}$ . Together with representative snapshots from their simulations shown in Figure 4, these data indicate that while both tautomers undergo a flipflop transition, they have distinct orientational preferences: TC_N_ prefers an approximately “horizontal” orientation, placing its four fused rings perpendicular to the membrane normal and exposing its phenolic diketone and amide groups to water; TC_Z_ adopts a predominantly “vertical” orientation and exposes its charged dimethylammonium and tricarbonyl to water. These behaviors can be explained by the distinct polarity profiles of the two tautomers: The deprotonation of the dimethylammonium significantly reduces the polarity of this moiety, rendering the TC_N_ tautomer nearly amphiphilic—four of its six hydrogen bond donors are placed on the phenolic diketone side and of the remaining two hydrogen bond donors, the hydroxyl from the tricarbonyl group readily forms an intramolecular hydrogen bond. Consistent with its polarity profile, the TC_N_ tautomer experiences a deep energy well (−4.9 kcal/mol) at the lipid–water interface, where it can expose its highly polar phenolic diketone side to water and bury the opposite, less polar side in the lipid tails. The TC_Z_ tautomer, in contrast, strongly favors exposing its positively charged dimethylammonium and negatively charged tricarbonyl to water. Since both of these charged components are near ring A (Figure 3 A), the “vertical” orientation that maximizes their exposure to water can only be achieved at the expense of burying the polar phenolic diketone group in lipid tails, which results in only a shallow energy well (−2.1 kcal/mol) for TC_Z_ at the lipid–water interface. The zwitterionic components in TC_Z_ also appear to elicit distinct hydrogen bond interactions and solvation shells from that of TC_N_, with the former solvated by ∼6 to 8 water molecules throughout its membrane permeation, compared to ∼3 to 5 water molecules accompanying the latter tautomer (Figure 3D–H). Overall, the charges carried by its zwitterionic components and the resulting greater requirement for solvation underlies the significantly higher energy barrier against the permeation of TC_Z_ compared to the neutral TC_N_.

**Figure 3:**
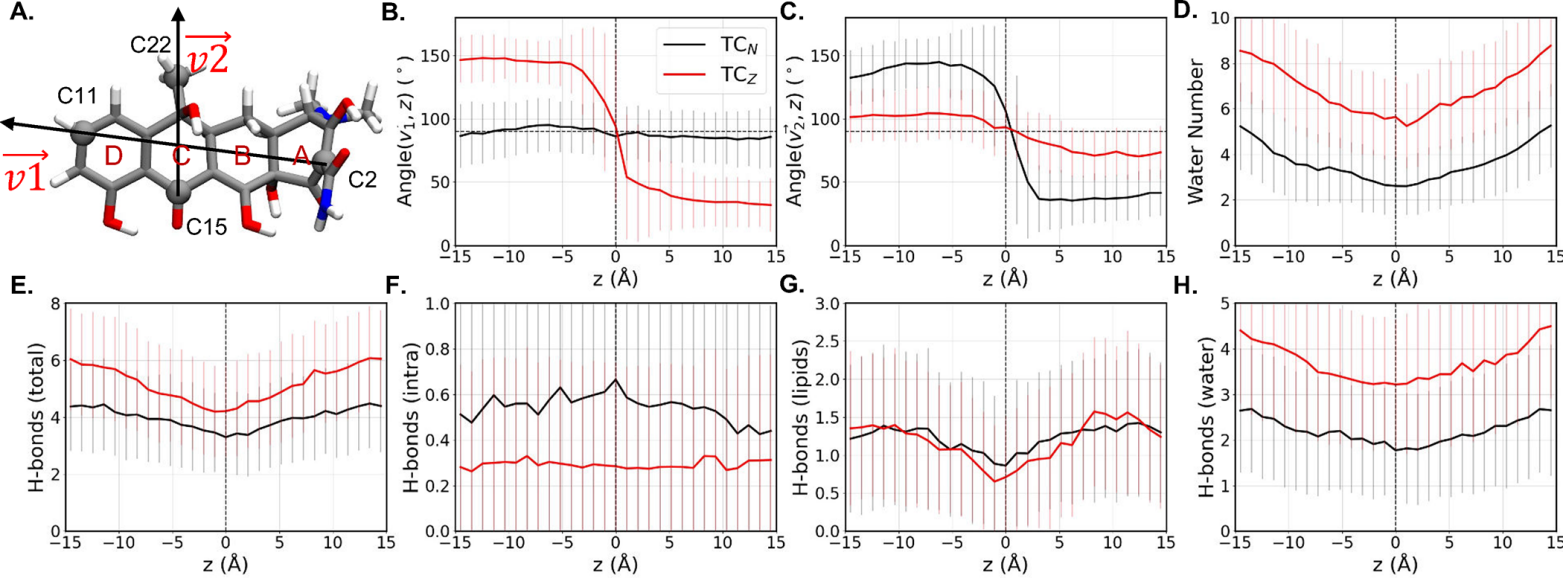
Orientation, the number of solvating water molecules and hydrogen bond (H-bond) interactions of TC during its membrane permeation. (A) Definition of ${\vec{v}}_{1}$ and ${\vec{v}}_{2}$ . (B, C) Angle between ${\vec{v}}_{1}$ (B) or ${\vec{v}}_{2}$ (C) and *z*-axis. (D) Number of water molecules within 2.4 Å of TC. (E-H) Number of total H-bonds (E), TC’s intramolecular H-bonds (F), and H-bonds between TC and POPC lipids (G) or water (H). Error bars represent the standard deviations.

**Figure 4:**
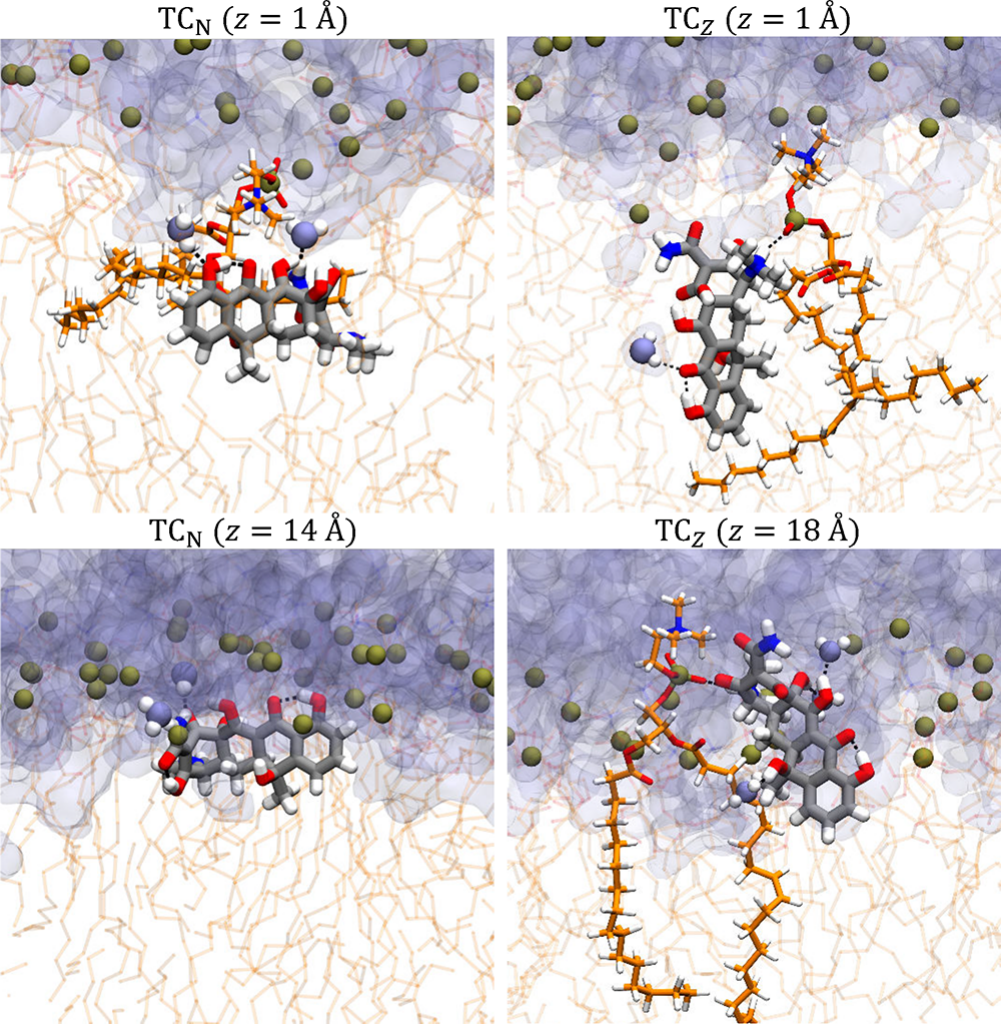
Representative snapshots of TC_N_ and TC_Z_ during membrane permeation. Hydrogen bonds within TC as well as those between TC and POPC or water molecules are shown in black dashed lines.

### Effective Permeability of TC at pH = 6 Is Dominated by TC_N_

To compute the effective permeability of TC at the weakly acidic condition (pH = 6) used in liposomal fluorescence experiments,^26,27^ we adopt the pH partitioning and BWAP protocols as given in Harris et al.^18^ Apart from membrane permeation PMFs and diffusivity profiles, both protocols require the ratios, or equivalently, the free-energy differences between various protonation microstates of the permeant molecule. As explained by Gunner et al.,^64^ a protonation macrostate is defined by the net charge of the molecule, while a microstate is defined by the specific protonation and tautomeric states of all its ionizable groups: For a simple molecule with a single ionizable group, each macrostate may contain only one microstate; for a complex molecule with multiple ionizable groups, a macrostate may contain multiple microstates that represent different tautomers of the molecule at the given net charge. With three ionizable groups, TC has four protonation macrostates characterized by a net charge of +1, 0, −1 and −2, respectively. Its complete ionization scheme showing all eight microstates is given in Figure 2C, where the superscripts indicate the charge carried by the tricarbonyl (A), the dimethylammonium (B) and the phenolic diketone group (C), respectively (Figure 1A). For instance, *A* ^0^ *B* ^0^ *C* ^0^ represents TC_N_, and *A* ^–^ *B* ^+^ *C* ^0^ corresponds to its tautomer TC_Z_. The remaining zero-net-charge tautomer *A* ^0^ *B* ^+^ *C* ^–^ is also a zwitterion and will be referred to as TC_Z*′*_ from hereon. In theory, both the pH partitioning protocol given in eq 2 and the BWAP treatment given in eq 3 should enumerate all microstates of a given molecule. In practice, calculating the membrane permeation free energy and diffusivity profiles for all microstates is neither feasible nor necessary, since only those microstates with significant fraction and/or high specific permeability make non-negligible contributions to *P*_eff_. At pH = 6, the fractions of the four macrostates of TC can be readily obtained from its three macroscopic p*K*_a_s, consistently found to be around 3, 7, and 9, respectively.^32,65,66^ In this work, we adopt the values reported by Leeson et al.,^65^ namely, p*K*_a1_ = 3.33, p*K*_a2_ = 7.75 and p*K*_a__3_ = 9.61, which are very similar to those reported by Stephens et al.^32^ and Qiang and Adams^66^ (see Table S1). As shown in Table 1, with a fraction of ∼98%, the zero-net-charge macrostate dominates at pH = 6, while the fractions of the +1 and −2 charged macrostates are three and 6 orders of magnitude smaller, respectively. The −1 charged macrostate is the second most populated state at this pH, which has a fraction of ∼1.74%. Since our calculations have revealed that the ±1 charged components in TC_Z_ drastically reduce its specific permeability, all charged macrostates of the antibiotic can be expected to have specific permeabilities at least as low as the zwitterionic TC_Z_. Therefore, in subsequent calculations, we assume that their specific permeabilities are capped by that of TC_Z_ (or equivalently, their membrane permeation PMFs lower-bounded by that of TC_Z_). Similarly, the alternative zwitterionic tautomer TC_Z*′*_ is assumed to have the same specific permeability as TC_Z_. As will be shown next, this assumption has negligible impact on the computed value of *P*_eff_, as long as the neutral TC_N_ remains significantly more permeable.

**Table 1:** Macrostates of TC and Their Fractions at pH = 6^a^

macrostate	*s* = 3	*s* = 2	*s* = 1	*s* = 0
net charge	+1	0	–1	–2
*f*_*s*_	2.10 × 10^–3^	TC_Z_: 9.65 × 10^–1^	1.74 × 10^–2^	4.30 × 10^–6^
		TC_N_: 1.40 × 10^–3^		
		TC_Z*′*_: 1.43 × 10^–2^		

a Instead of *f*_2_, fractions of the TC_Z_, TC_N_, and TC_Z*′*_ tautomers are shown.

We let *f*_*s*_ and *P*_*s*_ (*s* = 3, 2, 1, 0) represent the fractions and specific permeabilities of the +1, 0, −1, and −2 charged macrostates of TC, and *f*_N_, *f*_Z_ and *f*_Z*′*_ denote the fractions of the TC_N_, TC_Z_, and TC_Z*′*_ tautomers, respectively. It naturally follows that ∑_*s*_ *f*_*s*_ = 1 and *f*_2_ = *f*_N_ + *f*_Z_ + *f*_Z*′*_. Using the specific permeability of TC_Z_ as a proxy for all charged macrostates and the TC_Z*′*_ tautomer, we obtain the effective permeability of TC from the pH partitioning protocol as

$$P_{\mathrm{eff}}=\sum_{s}f_{s}P_{s}\approx (f_{0}+f_{1}+f_{Z}+f_{Z^{\prime }}+f_{3})P_{Z}+f_{N}P_{N}=(1-f_{N})P_{Z}+f_{N}P_{N}$$ 4

To determine *f*_N_, the ratios between the TC_N_, TC_Z_ and TC_Z*′*_ tautomers are needed. These pH-independent ratios cannot be determined from the macroscopic p*K*_a_s and instead require the microscopic acid dissociation constants, the negative log values of which are known as the microscopic p*K*_a_s (Figure 2). Although their experimental measurement is challenging, estimation can be made based on model compounds that are structural analogs of selected tautomers. For instance, since tetracycline methiodide (TCMI) hosts a permanent positive charge on its quaternary ammonium at site B,^67^ once it loses a proton on its tricarbonyl group A, the compound can be taken as an analog of *A* ^–^ *B* ^+^ *C* ^0^. The second macroscopic p*K*_a_ of TCMI has therefore been used by Leeson et al. as an estimate of *k*_13_, i.e., *pk*_13_ ≈ 7.80.^65^ Similarly, *k*_21_ and *k*_12_ have been estimated using another model compound as well as TC’s macroscopic p*K*_a_s: *pk*_21_ ≈ 5.97 and *pk*_12_ ≈ 8.8.^65^ Assuming that the acidity of the tricarbonyl group A is approximately the same in the neutral *A* ^0^ *B* ^0^ *C* ^0^ and the zwitterionic *A* ^0^ *B* ^+^ *C* ^–^, i.e., *pk*_31_ ≈ *pk*_21_, the ratio between the three tautomers can be readily estimated from thermodynamic cycles in the ionization scheme^64^—one such thermodynamic cycle involving *A* ^–^ *B* ^+^ *C* ^0^, *A* ^–^ *B* ^0^ *C* ^0^ and *A* ^0^ *B* ^0^ *C* ^0^ is highlighted in bold in Figure 2, from which *f*_N_/*f*_Z_ is found to be *k*_12_/*k*_21_ = 10^5.97–8.8^ = 10^–2.83^. Similarly, *f*_Z*′*_/*f*_Z_ is obtained from the thermodynamic cycle involving *A* ^–^ *B* ^+^ *C* ^0^, *A* ^–^ *B* ^+^ *C* ^–^ and *A* ^0^ *B* ^+^ *C* ^–^ as *f*_Z*′*_/*f*_Z_ = *k*_13_/*k*_31_ = 10^5.97–7.8^ = 10^–1.83^. The fractions of the three tautomers TC_N_, TC_Z_ and TC_Z*′*_ can then be calculated from their sum (*f*_2_). These values, in particular *f*_N_, are used to compute the effective permeability of TC via eq 4.

At pH = 6, the above calculation yields *P*_eff_ = 3.17 × 10^–5^ cm/s (log *P*_eff_ = −4.50), which is similar to the result of a PAMPA assay using a decane membrane with 10% soybean lecithin at pH = 6 (*P*_PAMPA_ ≈ 1.8 × 10^–5^ cm/s from Figure 3 of Yamauchi and Sugano^35^), but differs by approximately one log unit from log *P*_app_ = −5.86 measured using fluorescence assays conducted on POPC liposomes.^26,27^ Before we further discuss the nature of this deviation, we repeat the above calculation using the BWAP protocol: The PMF and diffusivity profiles of TC’s charged macrostates and the TC_Z*′*_ tautomer are again approximated by those of the TC_Z_ tautomer, yielding a weighted average potential *w*_m_(*z*) as

$$e^{-\beta w_{m}(z)}=\sum_{s}f_{s}e^{-\beta w_{s}(z)}\approx (1-f_{N})e^{-\beta w_{Z}(z)}+f_{N}e^{-\beta w_{N}(z)}$$ 5

where *w*_Z_(*z*) and *w*_N_(*z*) represent the original PMFs of TC_Z_ and TC_N_, respectively (Figure 2A). The PMF of TC_N_ can be shifted by Δ*G* = −1/β ln­(*f*_N_/*f*_Z_) to account for the free-energy cost for converting a TC_Z_ tautomer to TC_N_ in bulk solution at the given pH. The resulting shifted PMF of TC_N_ enables the direct comparison of its permeation energetics with that of TC_Z_, after the different fractions of the two tautomers are taken into consideration. As given in Table 2, calculation results based on the BWAP and the pH-partitioning schemes are indistinguishable. This behavior arises from the significantly higher free energy barrier of TC_Z_ than that of the shifted PMF of TC_N_, i.e., even after the lower fraction of the neutral tautomer has been taken into account, its contribution to *P*_eff_ remains dominant. As pointed out by Harris et al.,^18^ when one ionization state dominates *P*_eff_, both approaches yield similar numerical results.

**Table 2:** Specific Permeabilities of TC_N_ (*P*_N_), TC_Z_ (*P*_Z_), and the Effective Permeability (*P*_eff_) of TC at pH = 6 (Unit: cm/s)

*P*_N_	*P*_Z_	*P*_eff_ (pH-partitioning)	*P*_eff_ (BWAP)
2.22 × 10^–2^	1.63 × 10^–8^	3.17 × 10^–5^	3.17 × 10^–5^

The effective permeability of TC at pH = 6 depends minimally on the specific permeabilities of its charged macrostates of TC and that of the TC_Z*′*_ tautomer: setting these specific permeabilities to zero yields negligible reduction in *P*_eff_; similarly, increasing them by 3 orders of magnitude raises the computed *P*_eff_ by merely ∼1.7% (Table S2). Only when their specific permeabilities become 5 orders of magnitude greater, i.e., approaching that of *P*_N_, the computed *P*_eff_ increases by approximately 2.7 fold (Table S2). However, as its zwitterionic components have rendered the specific permeability of TC_Z_ much lower than its neutral tautomer, neither TC_Z*′*_ nor any charged macrostate of TC is expected to have specific permeability approaching that of TC_N_. The overall insensitivity of the computed *P*_eff_ to specific permeabilities of the charged macrostates is explained by the dominant fraction of the zero-net-charge macrostate at pH = 6, as well as the large specific permeability of TC_N_. Since *P*_N_ is 6 orders of magnitude greater than *P*_*Z*_, the neutral TC_N_ dominates *P*_eff_, even though its fraction is only ∼1/1000 that of TC_Z_. Indeed, as shown in Table S3, TC_N_ makes the dominant contribution to *P*_eff_ in acidic to basic environments with pH ranging from 1 to 10. However, we should add that this conclusion relies on the accuracy of the estimated *f*_N_/*f*_Z_ ratio, which is based on microscopic acid dissociation constants inferred from analog compounds. As discussed further below, the choice of these model compounds and the uncertainty in their macroscopic p*K*_a_ values directly affect the reliability of *f*_N_/*f*_Z_, and, hence, of the computed membrane permeability.

### Bilayer Patch Size Significantly Affects TC_N_ Permeation Energetics

While the TC_N_ tautomer faces a rather moderate energy barrier against its permeation, trajectory analysis reveals significant bilayer deformation as the molecule traverses the membrane. As shown in Figure S3, the monolayer occupied by TC_N_ “caves in” near the bilayer center, with deformation spanning over half the (*x*, *y*) plane. This observation motivates an investigation into the impact of bilayer patch size on TC permeability estimates, i.e., whether a minimum patch size is required to accommodate TC-induced membrane deformation and provide unbiased permeation energetics. Since TC_N_ dominates the effective permeability of the drug molecule over a wide range of pH, we focus our analysis on this tautomer. Toward this end, we performed additional PMF calculations for TC_N_ in bilayers containing 32, 64, and 256 POPC molecules. Together with the previously discussed 128-POPC system, these simulations enable a systematic assessment of patch-size effects. For comparison, we also simulated three other permeants—methanol, urea, and DDA— that differ in size and polarity (Table S4).^68^ The convergence of these PMF calculations is illustrated in Figures S4 and S5.

As shown in Figure 5 and Table S5, patch size exerts minimal influence on methanol, urea, and DDA, with permeation barriers differing by at most ∼9% across patch sizes. Specifically, methanol has a barrier of 3.4 kcal/mol in the 32-POPC system and 3.6 kcal/mol in the remaining three lipid patches—consistent with previous work reporting 3.5 ± 0.2 kcal/mol in a 100-POPC bilayer at 308 K.^59^ Urea exhibits no significant energy well, with a barrier ranging from 10.0 to 10.6 kcal/mol across the four systems, in line with the 9.4 kcal/mol barrier reported for a DMPC bilayer at 298.15 K.^6^ As the patch size increases, the barrier region (*w*(*z*) > 0) becomes slightly narrower—a trend also reported by Lee et al.^6^ Finally, DDA shows barriers between 7.4 and 8.1 kcal/mol, comparable to the 7.5 kcal/mol value observed for a 128-POPC bilayer at 308 K.^69^

**Figure 5:**
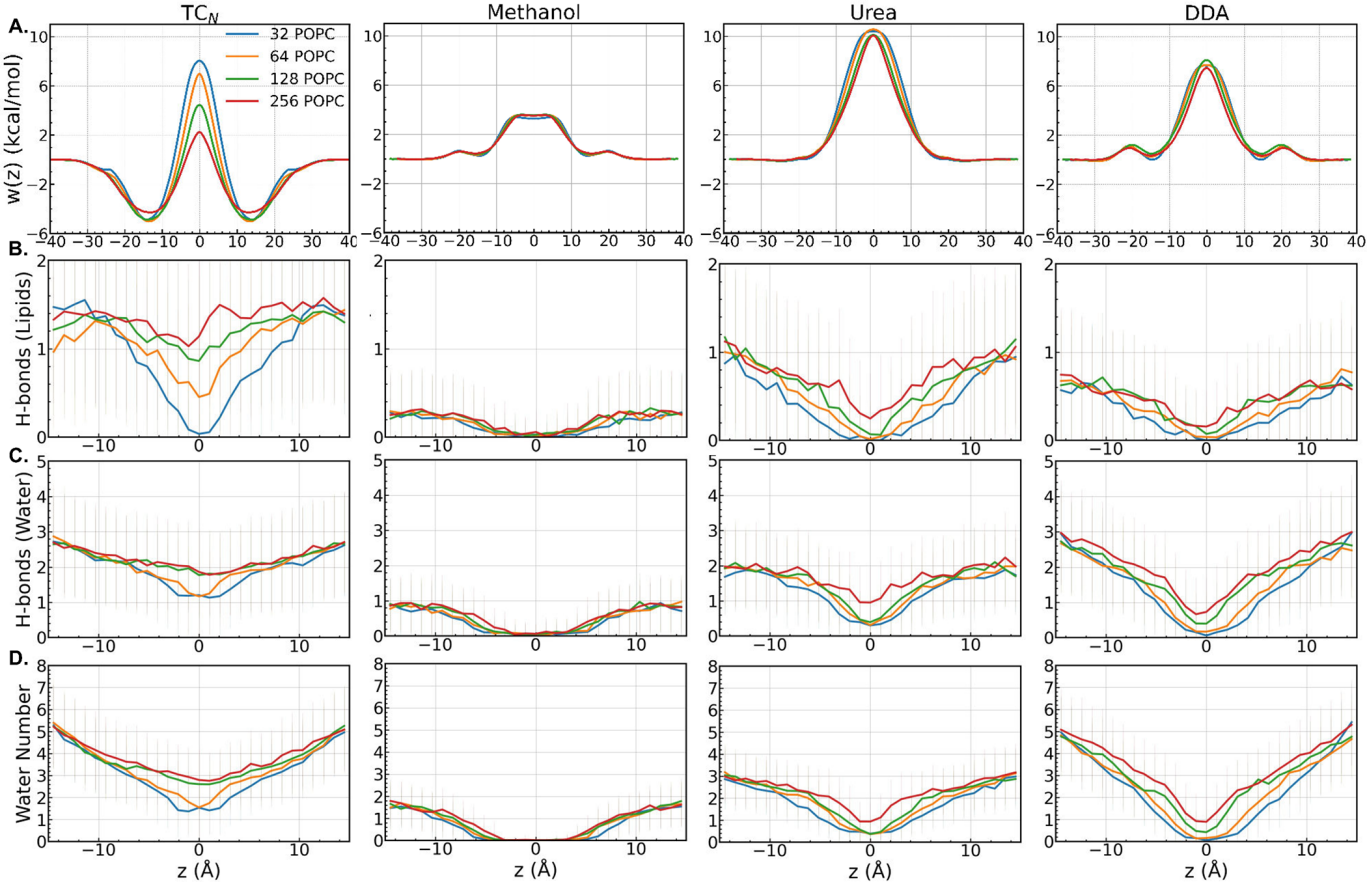
Membrane permeation PMFs and analysis of hydrogen-bond interactions and the number of solvating water molecules for TC_N_, methanol, urea, and DDA. (A) Symmetrized PMFs for each permeant. (B) Number of hydrogen bonds between each permeant and POPC. (C) Number of hydrogen bonds between each permeant and water. (D) Number of water molecules within 2.4 Å of each permeant. The error bars represent the standard deviations.

In contrast, the permeation energetics of TC_N_ exhibits a pronounced patch-size dependence. The energy barrier decreases from 8.1 kcal/mol in the 32-POPC patch to 7.0 kcal/mol in the 64-POPC patch, 4.4 kcal/mol in the 128-POPC system, and just 2.2 kcal/mol in the 256-POPC system. To understand this dependence, we compare the hydrogen-bonding interactions, hydration levels, and bilayer deformation across the four systems. TC_N_ forms on average 1.5 hydrogen bonds with POPC at the lipid–water interface (*z* = ±15 Å), exceeding values for methanol (0.2), urea (1.0), and DDA (0.6). These stronger interactions help explain why TC_N_, despite its size and Ro5 violation, has a lower barrier than urea or DDA. To minimize the energetic cost of membrane crossing, TC_N_ recruits hydrogen bonding partners from lipids or water, with the success of this recruitment varying with patch size. For example, at *z* = 0, TC_N_ maintains about 1 hydrogen bond in 128- and 256-POPC systems, but only about 0.5 in the 64-POPC patch, and virtually none in the 32-POPC system. Thermal undulations, which support such interactions, are constrained by simulation box size,^70^ making smaller bilayers effectively stiffer. Formation of hydrogen bonds at *z* = 0 demands substantial membrane deformation (Figure 4) and increased solvation deep in the bilayer (Figure 5). Smaller patches, like 32-POPC, resist such deformation, producing artificially high barriers. As shown in Figures S6 and S7, membrane thickness reduction is minimal for the 32-POPC bilayer, but evident in the larger systems. The inadequacy of the smallest 32-POPC patch in accommodating TC_N_ is further illustrated in the flip-flop behavior of the permeant. While an average orientation of ∼90° is recorded at *z* = 0 in all four bilayer patches (Figure S8), the distributions of the ${\vec{v}}_{2}$ tilt angle differ significantly: In both the 128-POPC and 256-POPC patches, and, to a lesser degree, the 64-POPC patch, TC_N_ mostly maintains a “horizontal orientation” where the angle between ${\vec{v}}_{2}$ and the *z*-axis is around 30° or 150° even in the bilayer center region (−1 < *z* < 1 Å); in sharp contrast, the same tilt angle is ∼90° in the 32-POPC bilayer (Figure S9), indicating that the smallest patch cannot support TC_N_ in its energetically favorable orientation. Along with the hydrogen-bonding patterns and membrane thickness profiles, this result explains the elevated energy barrier observed in the smaller bilayer systems for TC_N_.

While the smaller bilayers present artificially high barriers against TC_N_ permeation, further analysis suggests that the larger ones can be plagued by hysteresis. This issue is particularly evident once the patch size increases to 256 POPC. As illustrated in Figure 6, a TC_N_ molecule traversing the membrane from the lower to the upper leaflet may not undergo its flip-flop transition even at *z* ≈ 11.1 Å in the 256-POPC bilayer. The phenolic diketone side of TC_N_ continues to be solvated by water molecules that enter from the lower leaflet side. Its flip-flop transition only occurs at approximately *z* = 15 Å, after which the ${\vec{v}}_{2}$ tilt angle eventually switches to ∼30°, as typically observed at this location (Figure 6B). Such a lag in the change of TC_N_ orientation relative to its *z*-coordinate is a clear sign of hysteresis. As conformations where the membrane is minimally deformed and those with severe membrane deformation (Figure 6) all contribute to the force samples collected at *z* ≈ 11 – 15 Å, without proper weighting this *z*-range may be rendered less favorable than it actually is. Indeed, while the other three bilayers consistently reveal an energy well of approximately −4.9 kcal/mol at *z* = ±14 Å, the 256-POPC system shows a shallower well of −4.3 kcal/mol around this location. These values suggest that the impact of sampling hysteresis is the most severe in the largest bilayer patch, despite its apparent convergence (Figures S4 and S5). To further analyze the challenges posed by large bilayer patches, in Figure S10 we plot the *z*-locations of the four permeants relative to the center of the entire bilayer (*z*) against their *z*-locations relative to nearby lipids only (*z′*). As the bilayer patch size increases, the deviation between *z* and *z′* becomes evident and is the largest in the 256-POPC system with TC_N_. Consequently, the collective variable *z* becomes degenerate near the bilayer center in this system (and to a lesser degree, the 128-POPC system): a given *z* corresponds to multiple *z′*, which form two major branches that likely correspond to whether the permeant primarily deforms the upper or the lower leaflet. The degeneracy in *z* indicates that this “global” collective variable cannot always accurately reflect the “local” position of TC_N_ within a large bilayer patch. In such a system, the membrane conformation itself emerges as an additional slow degree of freedom. Given that the above behaviors arise from the inherent flexibility of large bilayers and the pronounced hydrogen-bonding capacity of TC_N_, merely increasing the simulation time is unlikely to resolve the associated hysteresis efficiently. Instead, the long-wavelength membrane deformations coupled with the permeant-lipid interactions suggest that multidimensional free-energy calculations, where the permeant orientation and/or even the membrane deformation serve as additional collective variable(s), may be needed. While such calculations are beyond the scope of the current study, for one-dimensional free-energy calculations conducted here, the 128-POPC and the 64-POPC patches both accommodate TC_N_-induced membrane deformation to various degrees without significant hysteresis observed in the largest 256-POPC bilayer. While we have focused on results obtained from the 128-POPC system, a *P*_eff_ of 3.72 × 10^–7^ cm/s (log *P*_eff_ = −6.43) is estimated by replacing the specific permeability of TC_N_ in eq 2 with that computed from the 64-POPC bilayer (while keeping other values the same as for the 128-POPC system). This estimated *P*_eff_ remains within one log unit of *P*_app_ measured using fluorescence assays,^26,27^ although it shows greater deviation from the result of a PAMPA assay.^35^ Taken together, our findings suggest that while a membrane that is too small should be avoided, e.g., the 32-POPC bilayer, potential hysteresis associated with large bilayer patches should also be closely monitored in permeation free-energy calculations of drug molecules with strong hydrogen bonding capacity.

**Figure 6:**
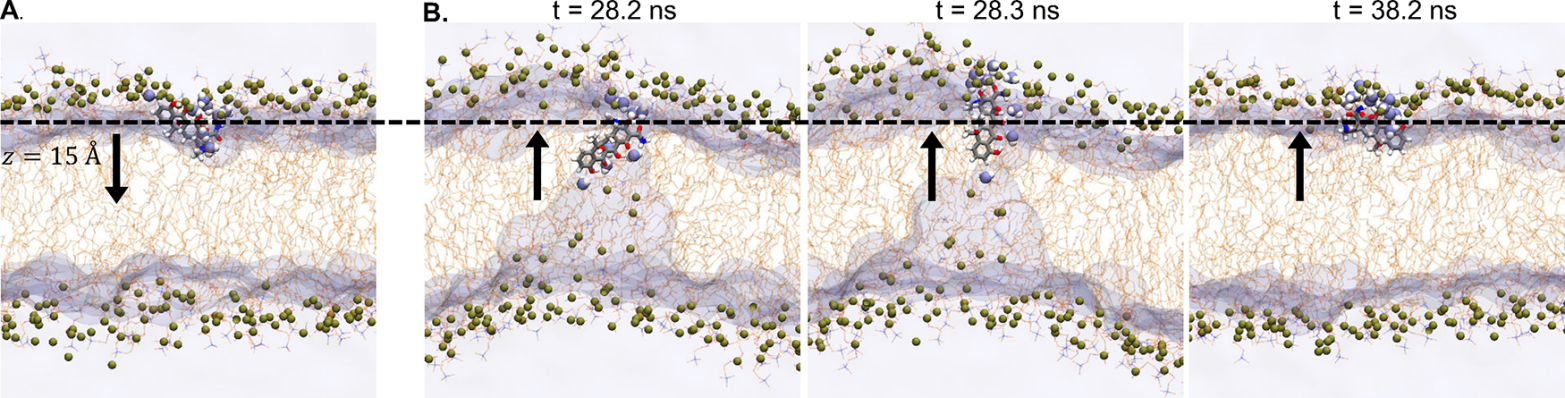
Snapshots of TC_N_ in the 256-POPC system. (A) TC_N_ entering the bilayer from the upper leaflet. (B) TC_N_ entering the bilayer from the lower leaflet. The location of TC_N_ and the angle between its ${\vec{v}}_{2}$ vector and the *z*-axis are 13.7 Å and 41° in (A), 11.1 Å and 138° (*t* = 28.2 ns), 15.0 Å and 99° (*t* = 28.3 ns), as well as 13.8 Å and 28° (*t* = 38.2 ns) in (B), respectively. Water is rendered as semitransparent surfaces, with those near TC_N_ highlighted in van der Waals representation. POPC molecules are shown in semitransparent licorice style, with their phosphorus atoms shown as tan spheres.

## Discussion

TC is one of the most widely used broad-spectrum antibiotics.^30^ Administered orally, it acts by inhibiting protein synthesis through targeting the bacterial ribosome. The amount of absorbed TC is directly proportional to its administered dose over a 10-fold range,^33^ indicating that TC absorption is a passive process.^36^ In both PAMPA and liposomal fluorescence assays, TC demonstrates moderate permeability on the order of 10^–6^ to 10^–5^ cm/s.^26,27,35^ These values are consistent with its rapid adsorption and good in vivo bioavailability,^33,34^ but appear paradoxical, given the six hydrogen-bond donors of the drug and its dominant zwitterionic population at neutral pH. Through permeation energetics and diffusivity calculations, we show that unlike the previously hypothesized cancellation-of-charge or ion-pair mechanism,^35,36^ the unexpectedly decent permeability of TC is attributed to its neutral tautomer TC_N_. While it has a fraction only ∼1/1000 that of the zwitterionic TC_Z_, its specific permeability is approximately 6 orders of magnitude greater. Consequently, the effective permeability of TC is dominated by TC_N_ over a wide range of pH values (Table S3).

To calculate the effective permeability of TC, one must combine the contributions of its various protonation and tautomeric states using protocols such as pH-partitioning or BWAP.^18^ Although they differ in their assumptions regarding how fast protonation is relative to membrane permeation, the two protocols yield indistinguishable numerical results for TC. This behavior arises from the significant difference between the PMF of TC_Z_ and the shifted PMF of TC_N_. As shown in Figure 2, even after their population fractions are taken into account, the permeation barrier faced by TC_N_, as revealed by its shifted PMF, is still much lower than TC_Z_. Therefore, whether the PMFs are first integrated to produce the specific permeabilities and then combined to yield the effective permeability as in the pH-partitioning protocol, or, combined first to yield an effective PMF and then used to compute *P*_eff_ as in the BWAP treatment, the numerical results are effectively indistinguishable. In other words, the population of the TC_Z_ tautomers near the bilayer center is sufficiently low such that their membrane permeation behavior—whether they cross as zwitterionic TC_Z_ or switch to neutral TC_N_—becomes insignificant in determining the value of *P*_eff_. Despite this numerical result, it is still of interest to ask, which one of these two processes, protonation or membrane permeation, is faster for TC? While an extensive analysis based on the transition path theory as carried out by Harris et al.^18^ is beyond the scope of this work, we note that the transition between TC_Z_ and TC_N_ does not involve any net release or uptake of protons. The switch between the two tautomers can proceed by transferring a proton from one ionizable group to another, i.e., the molecule serves simultaneously as its own proton donor and acceptor. Therefore, the low concentration of water within the membrane, which can slow down the protonation or deprotonation that involves net proton exchange, does not hinder this tautomeric transition. Given that TC only has a moderate permeability, it is plausible that the transition between its zwitterionic and neutral tautomers occurs faster than its membrane permeation.

As pointed out by Sezer and Oruç,^23^ results of a liposomal fluorescence assay may become unreliable if, instead of membrane permeation, the protonation/deprotonation of the permeant or the fluorescent dye becomes the rate-limiting step. At pH = 6, TC predominantly carries a zero net charge, and its macrostate carrying a −1 charge corresponds to the second highest fraction. In the fluorescence assay detailed previously,^8,27^ the molecule acts as a weak acid upon entering the liposome, i.e., TC crosses the membrane carrying zero net charge and then releases a proton. The pH-sensitive dye HPTS enclosed in the liposome then captures a proton, which in turn reduces its fluorescence signal. The rate of the fluorescence reduction (*k*) is used to estimate the apparent permeability of TC according to *P*_app_ = *kd*/6, where *d* ≈ 167.7 nm is the mean hydrodynamic diameter of the POPC liposomes.^27^ Following Harris et al.,^18^ we estimate the on-rate of protonation by *k*_on_ = *k*_0_ × 10^–pH^, where *k*_0_ = 10^10^ M^–1^s^–1^ is the intrinsic protonation rate for small molecules. With the liposomal fluorescence measurement performed at pH = 6, we have *k*_on_ = 10^10–6^ = 10^4^ s^–1^, which is taken to be the protonation rate for both HPTS and TC in the pH = 6 liposomal environment. The deprotonation rate *k*_off_ is estimated from *k*_*on*_ and the corresponding acid dissociation constant.^18^ Specifically, we use *k*_off_ = *k*_0_ × 10^–p*K*_*a*2_^ to estimate the rate of deprotonation of the zero-net-charge TC, obtaining *k*_off_ = 10^10–7.75^ = 10^2.25^ s^–1^. In comparison, the measured apparent permeability of TC (*P*_app_ = 10^–5.86^ cm/s) corresponds to an intrinsic rate *k* = 6*P*_app_/*d* ≈ 0.5 s^–1^. Since both *k*_on_ and *k*_off_ exceed *k* by orders of magnitude, the rate of fluorescence change cannot be reflecting that of TC deprotonation or HPTS protonation. Thus, unlike propranolol,^23^ the liposomal fluorescence assay result for TC should reflect the permeation dynamics of the drug molecule.

The many hydrogen bond donors and acceptors of TC present some unique challenges to its permeability calculation. For instance, several of its six hydrogen bond donors are part of the two ionizable groups of the molecule. With altogether three ionizable groups, the complete ionization scheme of TC involves eight microstates, which belong to four macrostates distinguished by the net charge carried by the molecule. Calculation of TC’s effective permeability requires not only the fraction of each macrostate, which can be readily computed using its macroscopic p*K*_a_s, but also the ratios between the tautomers that correspond to microstates in a given macrostate. These ratios depend on microscopic p*K*_a_s, which are considerably more difficult to estimate than macroscopic ones. While assignment based on analogous model compounds is a well-established strategy, the uncertainty associated with the p*K*_a_ measurement of these model compounds, and/or the choice of different model compounds may both affect the computed *P*_eff_. As an example, had p*k*_21_ been estimated from a different, slightly more dissimilar compound,^71,72^ an up to 0.5 log unit reduction in *P*_eff_ would be produced (Table S6).

Compared to the uncertainty in microscopic p*K*_a_ estimation, incorrect use of macroscopic p*K*_a_s presents an even greater danger: previous studies have attempted to assign the three macroscopic p*K*_a_s of TC to its three ionizable groups.^32,65,66^ However, while there is little ambiguity in assigning p*K*_a1_ ≈ 3.3 to the tricarbonyl group A, conflicting assignments have been made for the remaining two p*K*_a_s: Stephens et al.^32^ assigned p*K*_a2_ to the dimethylammonium group B and p*K*_a__3_ to the phenolic diketone group C, while the opposite assignment was given by Leeson et al.^65^ More recently, Qiang and Adams have shown that the latter two ionizable groups undergo deprotonation simultaneously during titration.^66^ As a result, p*K*_a2_ and p*K*_a3_ both have significant contributions from the dimethylammonium and the phenolic diketone groups, and neither of the two macroscopic p*K*_a_s can be unambiguously assigned to either group. Naively assigning the macroscopic *K*_a2_ and *K*_a3_ to the microscopic *k*_2_ and *k*_3_ (or in the reverse order) changes the computed *P*_eff_ by up to 3 orders of magnitude (Table S7), reflecting the substantial error introduced by incorrect macroscopic p*K*_a_ assignments. While the ionization scheme of TC appears more complex than a simple acid, other drug molecules, especially the bRo5 ones, may well present similarly (if not more) complex ionization schemes. Therefore, care should be taken to avoid the incorrect use of macroscopic p*K*_a_s in permeability calculation of these molecules.

Another challenge presented by TC is its strong hydrogen bonding capacity with lipid molecules. As it elicits surrounding lipids to help coordinating its many hydrogen bond donors and acceptors, membranes that cannot readily deform and cater to TC’s hydrogen bonding requests due to finite-size effects may artificially increase energy barriers against its permeation. As noted earlier, this result does not mean that larger membranes necessarily deliver superior performance in the permeability calculation of TC and other drugs with strong hydrogen bonding capacity. As demonstrated by the 256-POPC bilayer, significant hysteresis may occur in calculations using this large membrane and is unlikely to be efficiently resolved through increasing simulation time. The degeneracy in the collective variable *z* shown in Figure S10 should become ever more severe as the bilayer patch size further increases. Indeed, even without any permeant, larger bilayers exhibit greater thermal undulations as their effective bending modulus decreases for longer wavelengths,^70,73^ which are ultimately limited by the bilayer’s lateral dimensions. If a permeant molecule were to enter halfway into such a bilayer from a “peak” or “valley” position of its undulating surface, the collective variable *z*, measured relative to all lipid molecules in the membrane, may very well yield a value far from zero, even if the permeant is already at the midplane of the bilayer. While molecular simulations are unlikely to employ bilayer patches nearly as big, given the sensitivity of the estimated permeability to *w*(*z*), it may only take a relatively mild undulation to exacerbate the hysteresis already observed with the 256-POPC bilayer and the TC_N_ molecule. Overall, further studies with a broader range of patch sizes, sampling protocols, and/or multidimensional free-energy calculations—or, alternatively, a path search in multidimensional CV subspace combined with a one-dimensional free-energy calculation along the discovered path^74^—will be necessary to determine the onset of hysteresis and explore efficient mitigation strategies.

## Conclusions

Molecular simulations have long been employed to estimate the membrane permeability and probe the passive transport mechanism of drug or drug-like small molecules. The simulated drug molecules typically obey Lipinski’s “Rule-of-Fve”, a set of guidelines for screening compounds with desirable bioavailability. Whether these simulations can tackle more complex permeants, especially those beyond-Rule-of-Five drugs that violate more than one Ro5, remains largely unknown. With six hydrogen bond donors, the antibiotic TC violates one rule under the Ro5. While this still places it within the class of orally active drugs having “no more than one violation of the Ro5”, TC represents an ideal borderline case to explore the capacity of molecular simulations in tackling more complex and challenging permeants. In this work, we calculated the PMFs and diffusivity profiles governing the membrane permeation of two zero-net-charge tautomers of TC—the neutral TC_N_ and the zwitterionic TC_Z_—across 128-POPC lipid bilayers, and determined their intrinsic, pH-independent specific permeabilities using the ISD model. While the diffusivity profiles of TC_N_ and TC_Z_ are highly similar, their PMFs differ substantially, with the free-energy barrier of TC_N_ being 9 kcal/mol lower than that of TC_Z_. This large difference leads to a six-order-of-magnitude higher specific permeability of TC_N_ than TC_Z_. As a result, despite a fraction only ∼1/1000 that of the zwitterionic tautomer, the neutral TC_N_ dominates the effective permeability of TC over a wide pH range.

The computed effective permeability of TC at pH = 6 is 3.17 × 10^–5^ cm/s, which is close to the result of a PAMPA assay (1.8 × 10^–5^ cm/s) performed on a decane membrane with 10% soybean lecithin,^35^ but differs by approximately one log unit from that measured using a liposomal fluorescence assay (log *P*_app_ = −5.86).^26,27^ Overall, the approximately one log unit deviation between the computationally estimated *P*_eff_ and the experimentally measured *P*_app_ is comparable to the performance of permeability calculations for other drug and drug-like molecules.^9,11,17,20^ However, this result depends closely on the pH-independent ratio between TC_N_ and TC_Z_ (*f*_N_/*f*_Z_), which is estimated from the corresponding microscopic acid dissociation constants. Our calculations indicate that when incorrectly assigned macroscopic p*K*_a_s are used to estimate *f*_N_/*f*_Z_, *P*_eff_ may differ by orders of magnitude from that obtained using judiciously estimated microscopic p*K*_a_s. As ionization schemes can become particularly complex in drugs violating the Ro5 on the number of hydrogen bond donors and/or acceptors, extra care should be taken to avoid the incorrect use of macroscopic p*K*_a_s in their permeability calculations.

The observation of significant membrane deformation—up to half of the simulation box in the *xy* plane—prompted a systematic investigation of patch-size effects on the permeation energetics of TC_N_, given its dominant contribution to *P*_eff_. To provide a comparative perspective, we also examined three other molecules—methanol, urea, and DDA—which differ in size and physicochemical properties. PMFs were generated for bilayers composed of 32, 64, 128, and 256 POPC lipids and the results reveal a remarkable sensitivity of the energy barrier for TC_N_ to patch size, in stark contrast to the generally consistent PMFs observed for the other three permeants. Specifically, the free-energy barrier for TC_N_ decreases with increasing patch size, a trend attributable to the enhanced hydrogen bonding between TC_N_ and lipids facilitated by the greater membrane deformation achievable in larger bilayers. Such deformation in the largest 256-POPC bilayer produces significant hysteresis that challenges the convergence of the free-energy calculation. As a result, while extremely small bilayer patches should be avoided, potential hysteresis associated with large bilayer patches must be duly monitored, especially in permeability estimation of bRo5 drug molecules with strong hydrogen bonding capacity.

The above findings prompt broader reflection on the applicability and limitations of molecular simulation approaches to the study of complex permeants. While the agreement between simulation and experiment for the effective membrane permeability to TC is encouraging, the heavy reliance on a rare neutral species—one accounting for just a small fraction of the equilibrium population—raises questions about the generality and robustness of the mechanism. Does the observed behavior reflect a universal principle applicable to other complex, zwitterionic drugs, or is it an idiosyncrasy of the tautomeric landscape of TC? The pronounced sensitivity of the PMF to membrane patch size for TC_N_ also underscores the challenge of simulating the mechanically flexible bilayers, especially for large, polarizable permeants capable of remodeling their environment. These effects are rarely examined systematically, but may critically impact predictions of membrane permeabilities to drug molecules from first principles. As computational methods continue to mature, it will be essential to recognize that achieving numerical agreement with experimental observables does not guarantee mechanistic accuracy—particularly when conclusions hinge on rare or extreme configurations.

## Supplementary Material


