# Supplementary material for: Probing Passive Permeation of Tetracycline: Are Simulations Ready for beyond-Rule-of-Five Drug Permeability Calculation?
Source: J Phys Chem B. 2025 Oct 3;129(41):10810–23. doi: 10.1021/acs.jpcb.5c05445 (PMC12536400; doi:10.1021/acs.jpcb.5c05445)
Supplement: Supplementary file 1 [file jp5c05445_si_001.pdf]

Supporting Information Available

Probing Passive Permeation of Tetracycline: Are Simulations Ready for beyond-Rule-of-Five Drug Permeability Calculation?

Yajing Qi[1], Christophe Chipot[2,3,4], Yi Wang[1]

[1]Department of Physics, The Chinese University of Hong Kong, Shatin, Hong Kong SAR, China

[2]Laboratoire International Associé Centre National de la Recherche Scientifique et University of Illinois at Urbana-Champaign, Unité Mixte de Recherche n°7019, Université de Lorraine, 54506 Vandœuvre-lès-Nancy, France

[3]Theoretical and Computational Biophysics Group, Beckman Institute, and Department of Physics, University of Illinois at Urbana-Champaign, Urbana, IL 61801, USA

[4]Department of Biochemistry and Molecular Biology, University of Chicago, Chicago, IL 60637, USA

Table S1: Macro pK_a values of TC reported by three previous studies.

Reference	pK_a1	pK_a2	pK_a3
Stephens, et al.[32]	3.30	7.68	9.69
Leeson et al.[65]	3.33	7.75	9.61
Qiang and Adams[66]	3.32	7.78	9.58

Table S2: The effective permeability (P_{eff}) of TC and the contribution from the neutral tautomer ($\frac{P_N f_N}{P_{\text{eff}}}$) as the specific permeability (P_s^{other}) of species other than TC_Z and TC_N (all microstates carrying a net charge and the neutral $TC_{Z'}$ tautomer) changes. The calculation was performed at pH = 6 using the pH-partitioning scheme.

P_{s} (others)	P_{eff} (cm/s)	$\frac{P_N f_N}{P_{\text{eff}}}$ (%)
0	3.17×10^{-5}	99.95%
P_Z	3.17×10^{-5}	99.95%
$10P_Z$	3.17×10^{-5}	99.93%
$100P_Z$	3.17×10^{-5}	99.78%
$1000P_Z$	3.22×10^{-5}	98.24%
$10000P_Z$	3.72×10^{-5}	85.11%
$100000P_Z$	8.69×10^{-5}	36.44%

Table S3: The effective permeability of TC (P_{eff}) at various pH values obtained using the pH-partitioning scheme (unit: cm/s).

pH	P_{eff} (cm/s)	$\frac{P_N f_N}{P_{\text{eff}}}$ (%)
0	3.14×10^{-8}	48.0%
1	1.67×10^{-7}	90.2%
2	1.50×10^{-6}	98.88%
3	1.03×10^{-5}	99.84%
4	2.66×10^{-5}	99.94%
5	3.16×10^{-5}	99.95%
6	3.17×10^{-5}	99.95%
7	2.74×10^{-5}	99.94%
8	1.14×10^{-5}	99.86%
9	1.41×10^{-6}	98.84%
10	6.88×10^{-8}	76.27%
11	1.70×10^{-8}	4.17%

Table S4: Physicochemical properties of permeants investigated in this work as given by Chemicalize.[68]

	Methanol CH_3OH	Urea CH_4N_2O	DDA $C_{10}H_{13}N_5O_2$	Tetracycline $C_{22}H_{24}N_2O_8$
MW	32	60	235	444.4
nHDon	1	2	2	6
nHAcc	1	1	6	9
nC	1	1	10	22
nAR	0	0	2	4
ClogP	-0.52	-1.36	-0.48	-3.50

Table S5: Simulation times of WTM-eABF calculations, the free energy barriers and wells of four permeants across POPC bilayers with different sizes.

Permeant	Bilayer	Time (μs)	$w_{\text{peak}}(z)$ (kcal/mol)	$w_{\text{valley}}(z)$ (kcal/mol)	Valley Position (Å)
Methanol	32	3.0	3.4 ± 0.0	-0.0 ± 0.0	± 28.5
	64	3.0	3.6 ± 0.2	-0.0 ± 0.2	± 30.9
	128	4.0	3.6 ± 0.1	-0.0 ± 0.1	± 37.7
	256	3.5	3.6 ± 0.2	-0.0 ± 0.2	± 32.3
Urea	32	2.9	10.4 ± 0.0	-0.2 ± 0.1	± 24.7
	64	3.0	10.6 ± 0.2	-0.1 ± 0.2	± 24.5
	128	3.0	10.1 ± 0.1	-0.1 ± 0.1	± 25.5
	256	2.3	10.0 ± 0.1	-0.1 ± 0.1	± 24.5
DDA	32	3.0	7.7 ± 0.1	-0.1 ± 0.1	± 29.1
	64	6.0	7.7 ± 0.1	-0.1 ± 0.1	± 28.7
	128	3.0	8.1 ± 0.0	-0.1 ± 0.0	± 31.3
	256	3.3	7.4 ± 0.1	-0.0 ± 0.1	± 35.5
TC_N	32	5.0	8.1 ± 0.5	-4.9 ± 0.4	± 14.3
	64	9.0	7.0 ± 0.6	-5.0 ± 0.6	± 13.7
	128	8.0	4.4 ± 0.1	-4.9 ± 0.2	± 14.1
	256	6.8	2.2 ± 0.7	-4.3 ± 0.7	± 13.5
TC_Z	128	9.0	13.6 ± 0.2	-2.1 ± 0.5	± 18.1

Table S6: Effective permeability (P_{eff}) of TC according to the pH-partitioning scheme as a different model compound, 4-Dedimethylamino sancycline (Col-3), is used to estimate pk_{21}. The macroscopic p$K_{\mathrm{a}1}$ values of Col-3 given by Chemicalize[68] and Pinsuwan et al.[71,72] are used.

pk_{21}	P_{eff} (cm/s)	log P_{eff}
5.82[68]	2.25×10^{-5}	-4.65
5.64[71]	1.50×10^{-5}	-4.83
5.40[72]	8.64×10^{-6}	-5.06

Table S7: Effective permeability (P_{eff}) of TC according to the pH-partitioning scheme obtained by assigning the macroscopic pK_{a2} and pK_{a3} to the microscopic pk_2 and pk_3, respectively (or in the reverse order). The macroscopic p$K_{\text{a1}} = 3.33$ is assigned to pk_1 in both cases. The fractions of TC_N, TC_Z, and $\text{TC}_{\text{Z}'}$ are estimated using thermodynamic cycles involving $A^0B^+C^0$, $A^-B^+C^0$, $A^0B^0C^0$ and $A^0B^+C^-$ in Fig. 2.

Assignment	P_{eff} (cm/s)	$\log P_{\text{eff}}$
p$k_2 = 7.75$, p$k_3 = 9.61$	8.43×10^{-7}	-6.07
p$k_2 = 9.61$, p$k_3 = 7.75$	2.79×10^{-8}	-7.55

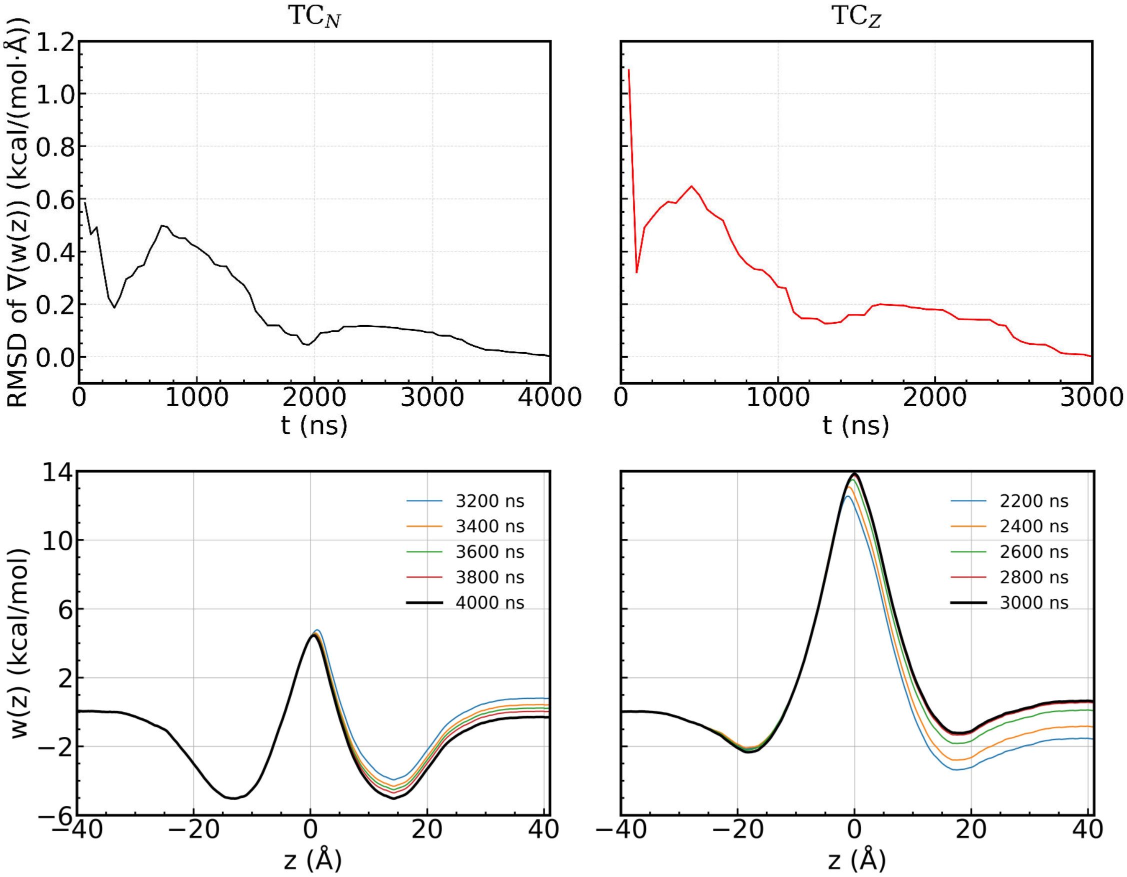

Figure S1: Convergence of the PMF calculation of TC_Z and TC_N in a 128-POPC bilayer assessed by the RMSD of the potential gradients $\nabla w(z)$ and the evolution of the PMF $w(z)$ as the simulation time increases.

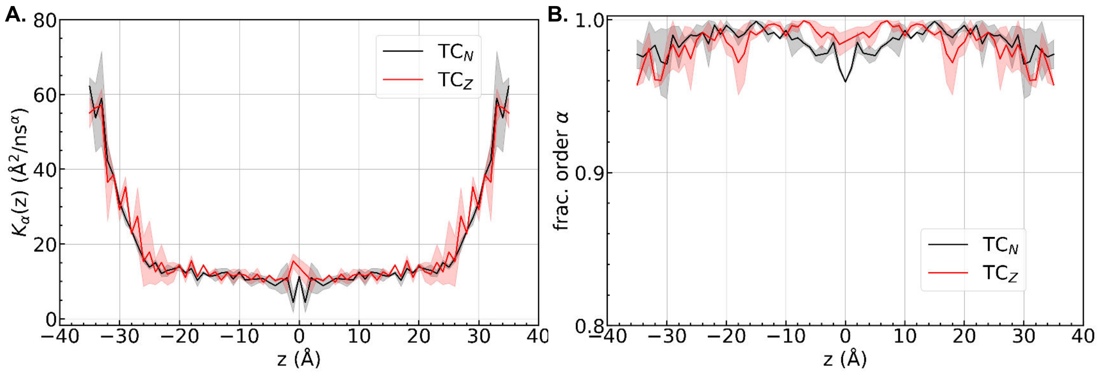

Figure S2: (A) The fractional diffusivity profiles of TC_Z and TC_N in a 128-POPC bilayer. (B) Profiles of the estimated fractional order α.

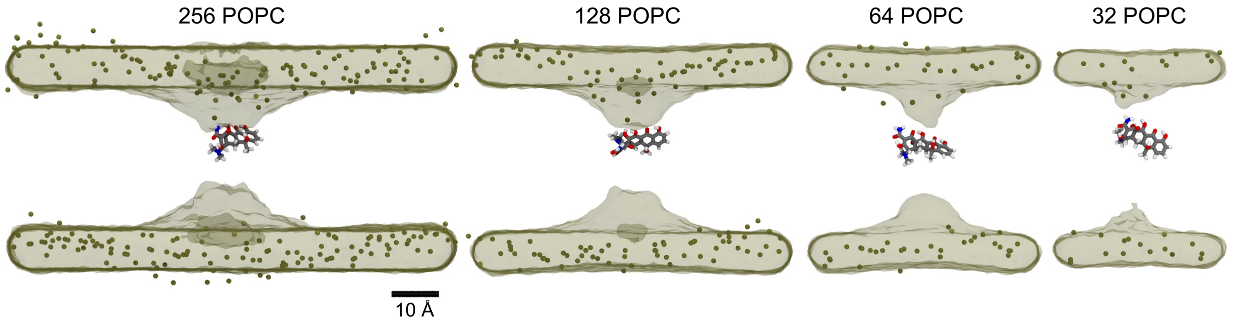

Figure S3: Lipid phosphorus atom occupancy at an isovalue of 0.02 for TC_N in POPC bilayers of different sizes, calculated from trajectories with TC_N in the region $-5 < z < 5$ Å.

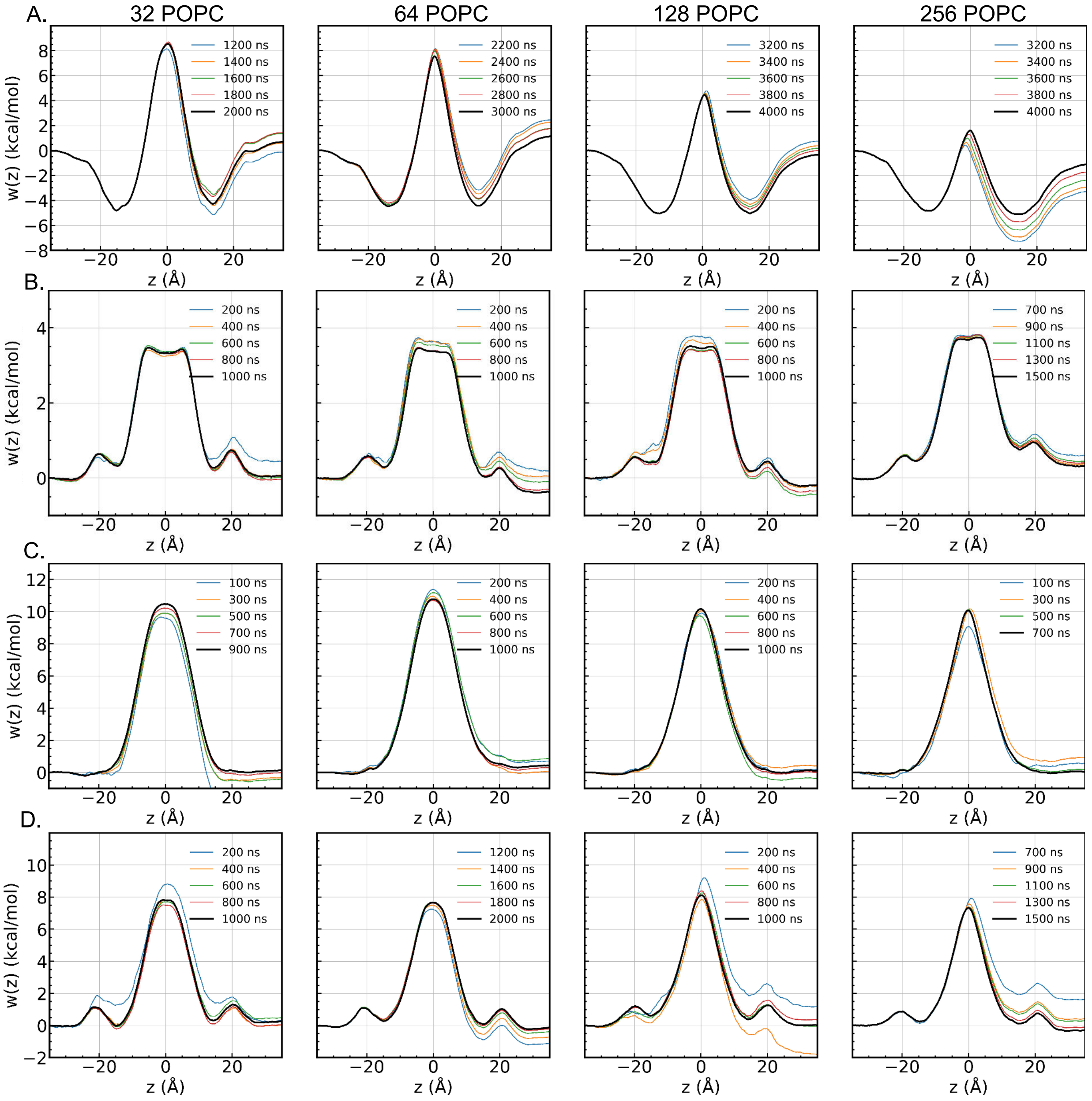

Figure S4: Convergence of PMF profiles for TC_N (A), methanol (B), urea (C), and DDA (D) in POPC bilayers of varying sizes.

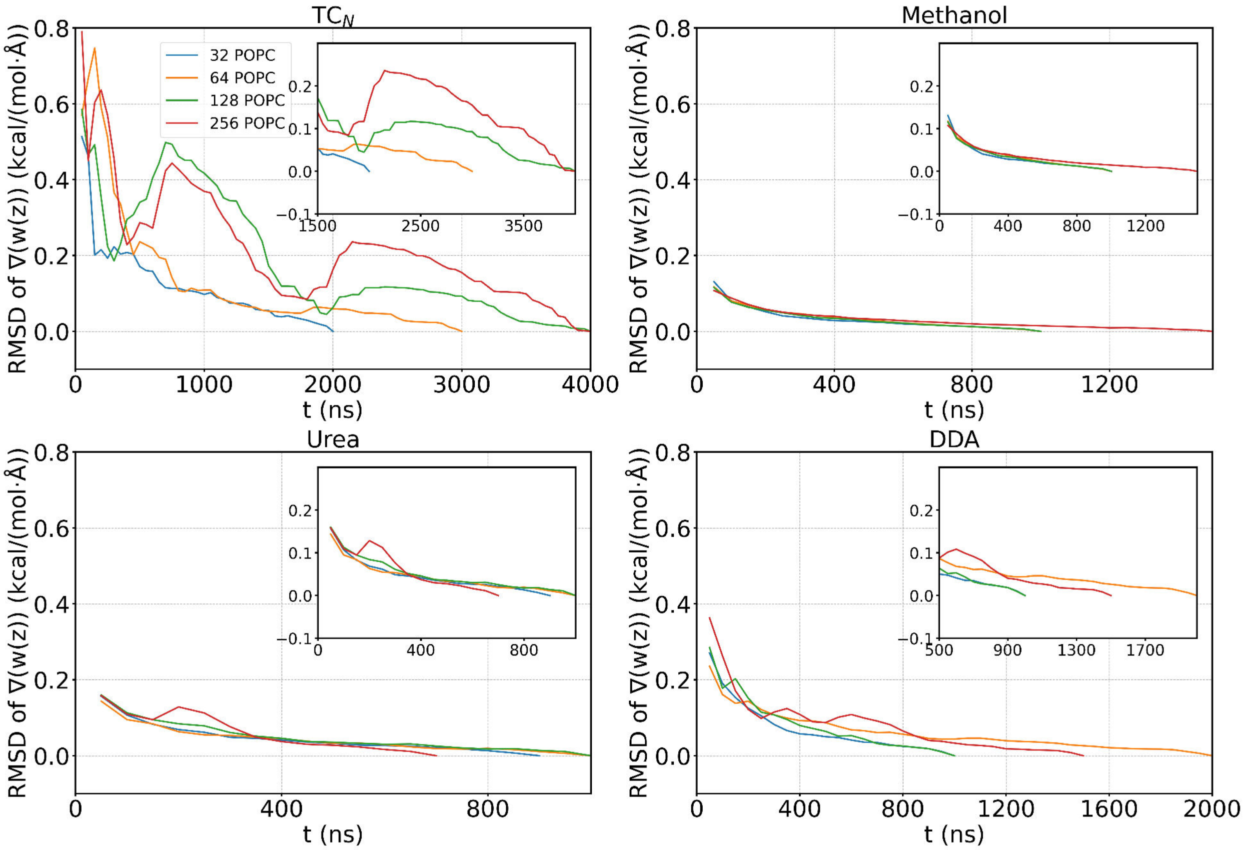

Figure S5: RMSD of the potential gradients ($\nabla w(z)$) as a function of simulation time t for the four permeants in membrane systems containing 32, 64, 128, and 256 POPC, respectively.

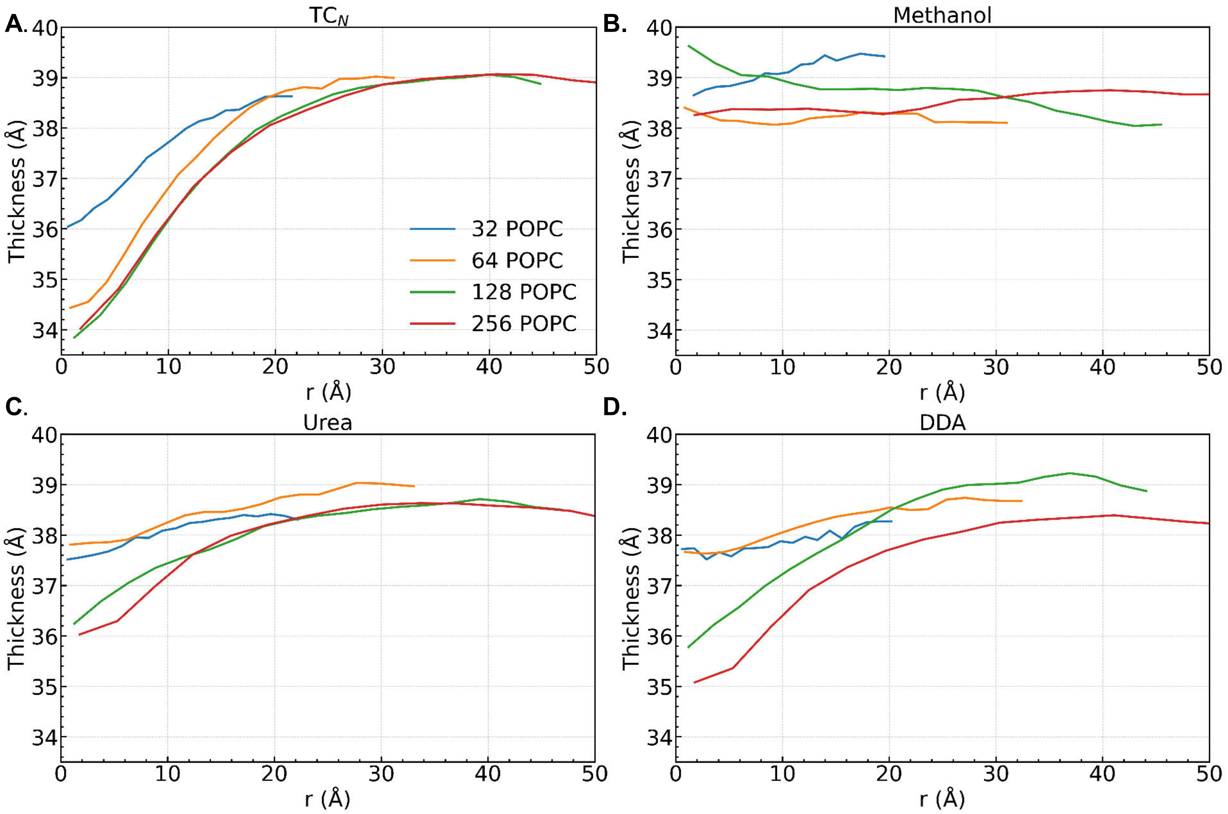

Figure S6: Membrane thickness as a function of the lateral distance in the xy plane from the center of each permeant (r) in POPC bilayers of different sizes, shown for (A) TC_N, (B) methanol, (C) urea, and (D) DDA.

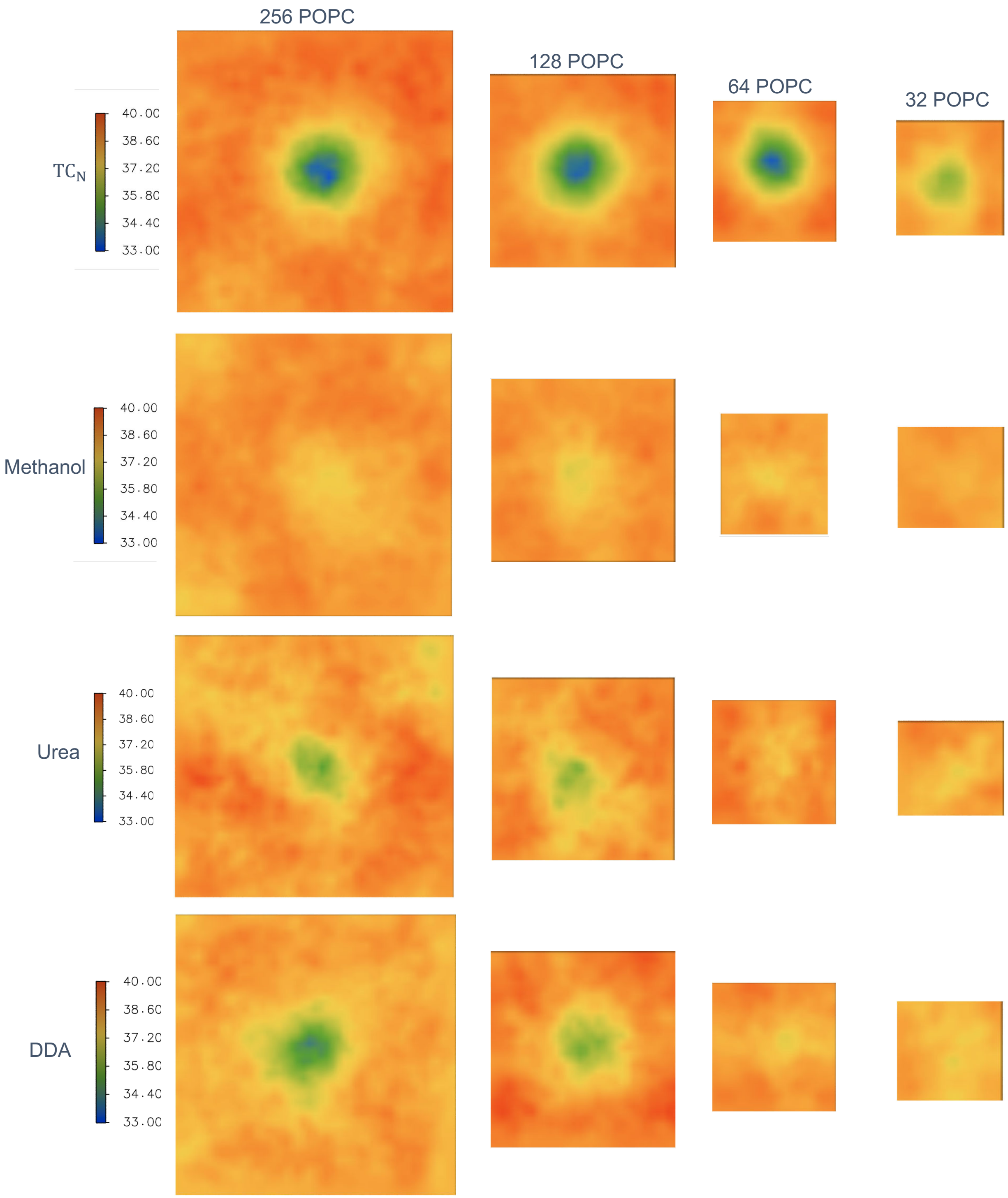

Figure S7: The membrane thickness heatmaps of TC_N, methanol, urea, and DDA when the permeants are located within the region $-5 < z < 5$ in POPC bilayers of different sizes.

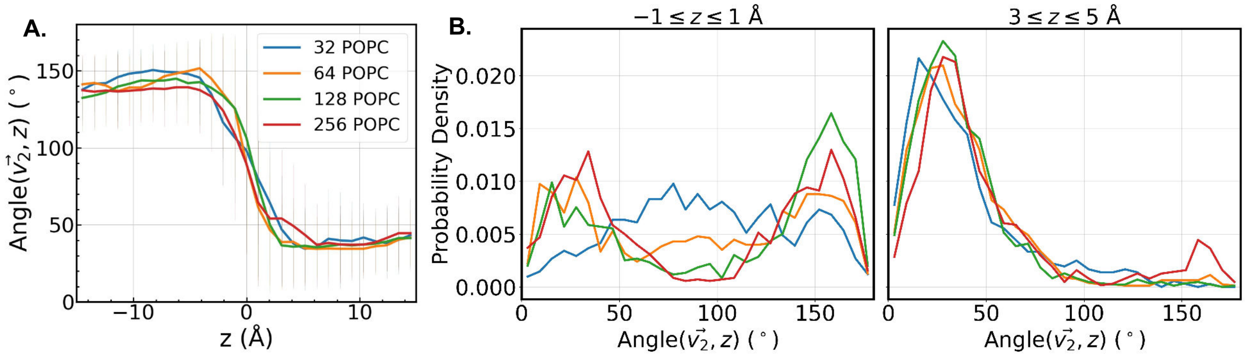

Figure S8: (A) Orientation of TC_N, as indicated by the angle between its vector $\vec{v_2}$ and the z-axis, with error bars representing standard deviations. (B) Probability density distributions of the orientation of TC_N in the regions $-1 \leq z \leq 1$ Å and $3 \leq z \leq 5$ Å of POPC bilayers with different sizes.

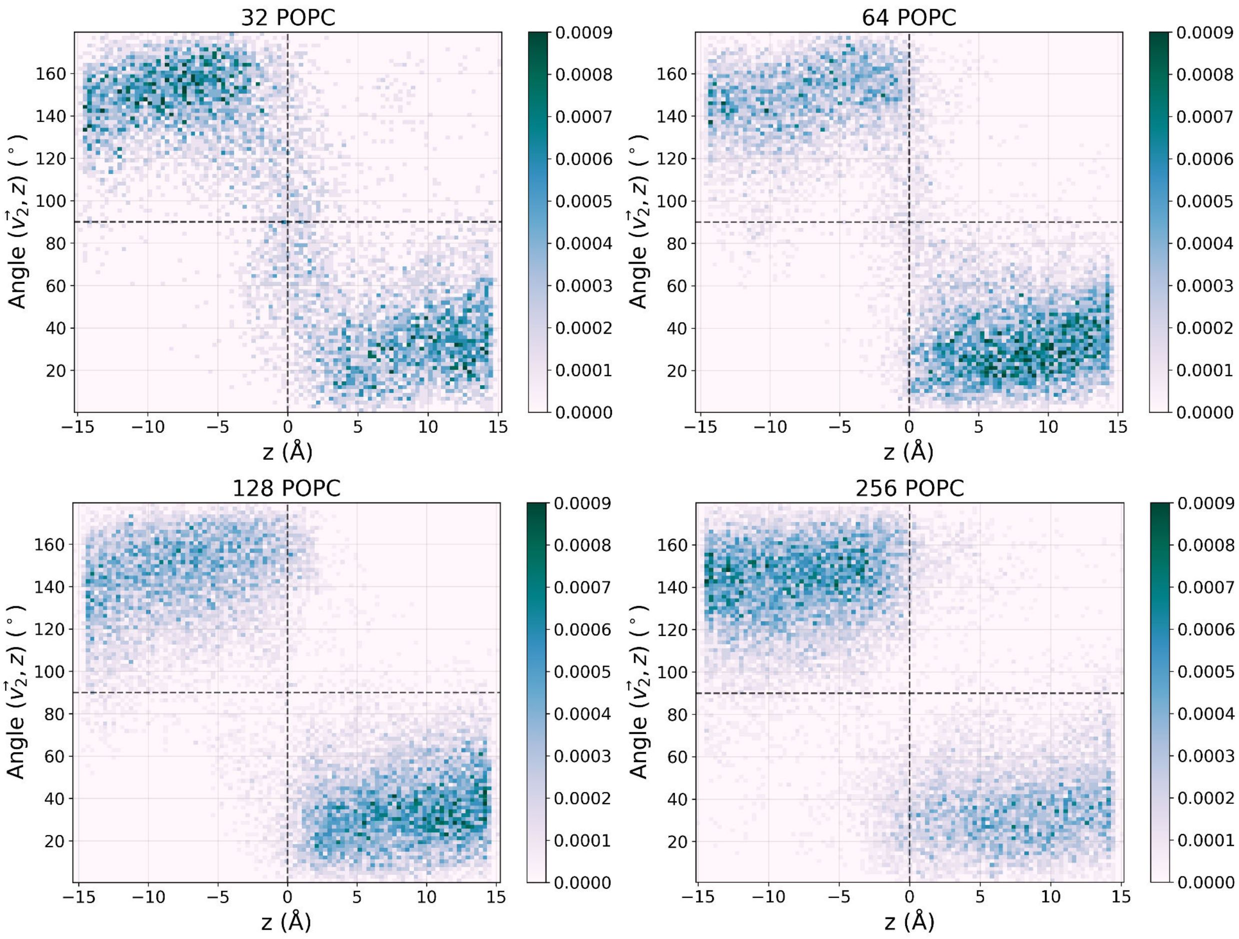

Figure S9: Probability density maps of the angle between $\vec{v_2}$ and the z-axis of TC_N in bilayers containing 32, 64, 128, and 256 POPC, respectively.

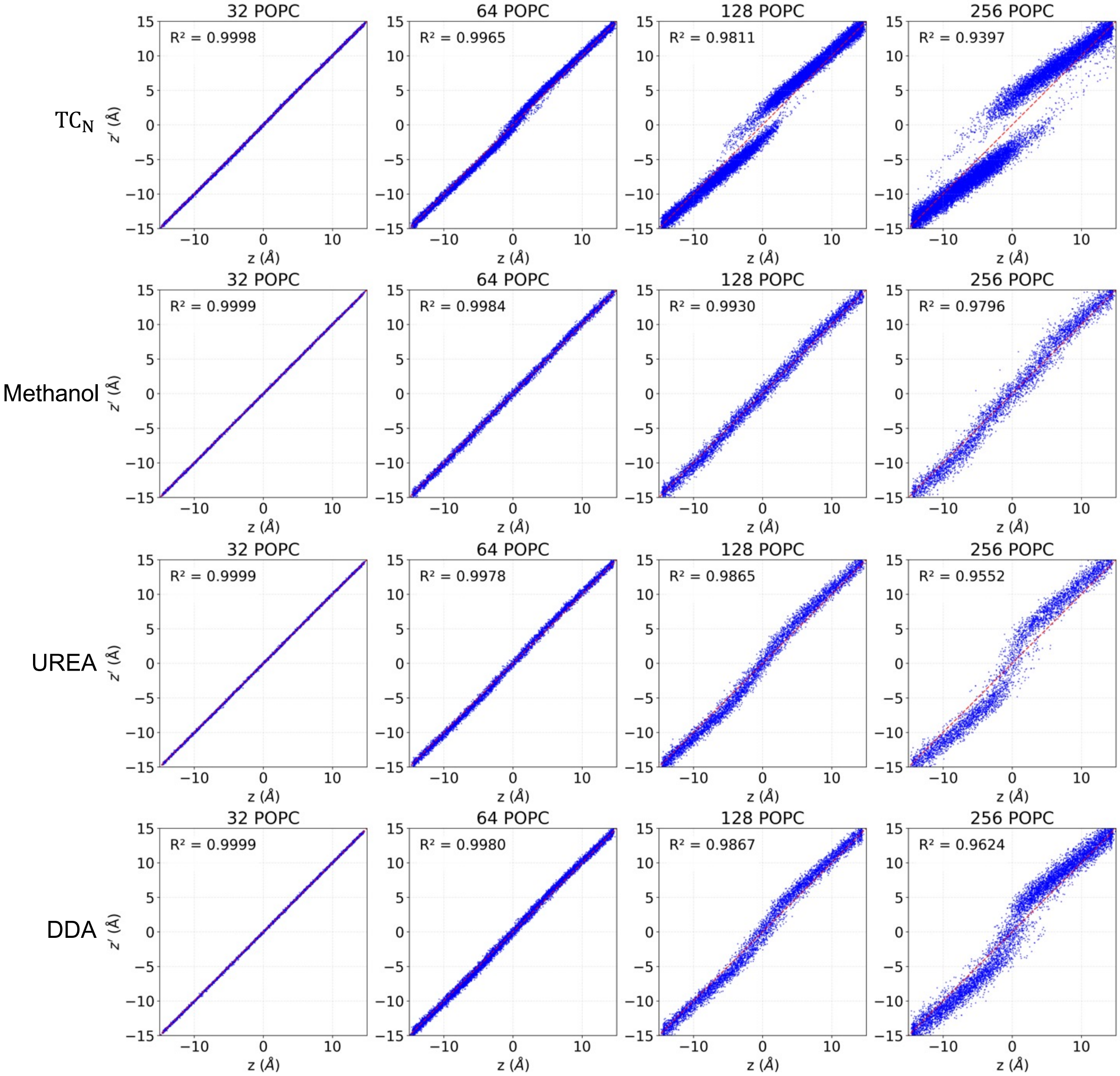

Figure S10: The z-coordinates of TC$_\mathrm{N}$, methanol, urea, and DDA relative to the center of mass of all lipid phosphorus atoms (z), or, only those lipid phosphorus atoms with a lateral distance less than 20 Å in the xy plane from the center of each permeant (z'). The dashed red line denotes the identity $z = z'$, while R^2 stands for the coefficient of determination from a linear fit of z' onto z.
